# Deciphering meiotic chromatin organization by SYCP3

**DOI:** 10.1093/nar/gkaf460

**Published:** 2025-06-09

**Authors:** Shimeng Guo, Yiran Zhang, Caifeng Fei, Xiaozhao Liu, Wei Xia, Mengcheng Luo, Gonghong Wei, Weibing Qin, Chengliang Xiong, Honggang Li, Ying Yin, Ximiao He, Li-quan Zhou

**Affiliations:** Institute of Reproductive Health & School of Basic Medicine, Tongji Medical College, Huazhong University of Science and Technology, Wuhan, Hubei 430030, China; Chongqing Key Laboratory of Human Embryo Engineering and Precision Medicine, Center for Reproductive Medicine, Women and Children’s Hospital of Chongqing Medical University, Chongqing 400016, China; Institute of Reproductive Health & School of Basic Medicine, Tongji Medical College, Huazhong University of Science and Technology, Wuhan, Hubei 430030, China; Institute of Reproductive Health & School of Basic Medicine, Tongji Medical College, Huazhong University of Science and Technology, Wuhan, Hubei 430030, China; Department of Physiology, School of Basic Medicine, Tongji Medical College, Huazhong University of Science and Technology, Wuhan, Hubei 430030, China; Center for Genomics and Proteomics Research, School of Basic Medicine, Tongji Medical College, Huazhong University of Science and Technology, Wuhan, Hubei 430030, China; Hubei Key Laboratory of Drug Target Research and Pharmacodynamic Evaluation, Huazhong University of Science and Technology, Wuhan, Hubei 430030, China; Institute of Reproductive Health & School of Basic Medicine, Tongji Medical College, Huazhong University of Science and Technology, Wuhan, Hubei 430030, China; Department of Tissue and Embryology, School of Basic Medical Sciences, Wuhan University, Wuhan, Hubei 430072, China; Fudan University Shanghai Cancer Center & MOE Key Laboratory of Metabolism and Molecular Medicine and Department of Biochemistry and Molecular Biology of School of Basic Medical Sciences, Shanghai Medical College of Fudan University, Shanghai 200032, China; NHC Key Laboratory of Male Reproduction and Genetics, Guangdong Provincial Reproductive Science Institute, Guangzhou 510600, China; Institute of Reproductive Health & School of Basic Medicine, Tongji Medical College, Huazhong University of Science and Technology, Wuhan, Hubei 430030, China; Institute of Reproductive Health & School of Basic Medicine, Tongji Medical College, Huazhong University of Science and Technology, Wuhan, Hubei 430030, China; Department of Physiology, School of Basic Medicine, Tongji Medical College, Huazhong University of Science and Technology, Wuhan, Hubei 430030, China; Center for Genomics and Proteomics Research, School of Basic Medicine, Tongji Medical College, Huazhong University of Science and Technology, Wuhan, Hubei 430030, China; Hubei Key Laboratory of Drug Target Research and Pharmacodynamic Evaluation, Huazhong University of Science and Technology, Wuhan, Hubei 430030, China; Department of Physiology, School of Basic Medicine, Tongji Medical College, Huazhong University of Science and Technology, Wuhan, Hubei 430030, China; Center for Genomics and Proteomics Research, School of Basic Medicine, Tongji Medical College, Huazhong University of Science and Technology, Wuhan, Hubei 430030, China; Hubei Key Laboratory of Drug Target Research and Pharmacodynamic Evaluation, Huazhong University of Science and Technology, Wuhan, Hubei 430030, China; Institute of Reproductive Health & School of Basic Medicine, Tongji Medical College, Huazhong University of Science and Technology, Wuhan, Hubei 430030, China

## Abstract

Chromatin structure during meiosis is different from somatic cells due to the assembly of the synaptonemal complex between homologous chromosome axes. However, genome-wide organizing principles of this meiosis-specific multiprotein complex remain mysterious despite intensive super-resolution imaging analysis. Here, we profiled chromatin occupancy of SYCP3, the key chromatin organizer of synaptonemal complex, in mouse spermatocytes, and showed its enrichment at open chromatin regions. Moreover, SYCP3 occupancy was largely inherited from the leptotene to pachytene stage, facilitated by transcription and fibrous assembly, and was enriched at specific SINE repeats. We also identified SYCP1-occupied regions mainly as a subpopulation of SYCP3-occupied regions with high cohesin enrichment. Collectively, our results demonstrate genome-wide profiling of SYCP3 in mouse meiosis and reveal that its occupancy is a dynamic process modulated by chromatin-related events.

## Graphical Abstract

**Graphical Abstract:**
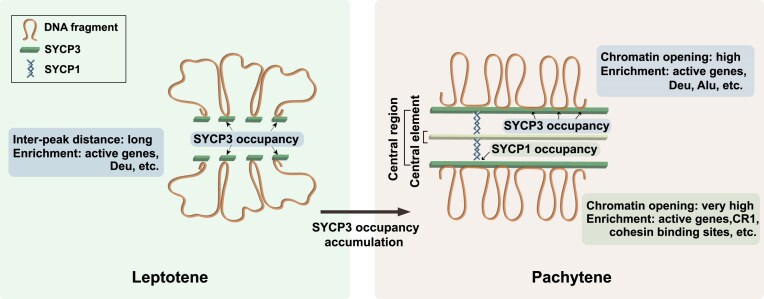

## Introduction

Meiosis is a critical step during the process of gametogenesis to form haploid cells, which finally develop into functional gametes. The synaptonemal complex (SC) is a meiosis-specific protein assembly that bridges homologous chromosomes to establish the architecture of the meiotic chromosome [1]. SC is composed of two parallel lateral elements (LEs), which associate with chromosomes, a central element (CE), and transverse filaments (TFs), which connect LE and CE [2]. SC precursor begins to appear at the leptotene stage of meiotic prophase I, and SC structure is complete by the pachytene stage. Synaptonemal complex protein 3 (SYCP3) is a highly extended helical tetrameric protein that has direct interaction with DNA as early as in the leptotene stage and self-assembles into filamentous structures to shape meiotic chromosome axes [3]. Male mice with a null mutation of SYCP3 failed to form functional LEs and SCs, resulting in developmental arrest during meiotic prophase [4]. SYCP3/2 are the major constituents of the LE, and the CE contains SYCE1-3 and TEX12, while SYCP1 forms TF through self-assembly with its two terminal regions located in CE and LE, respectively [5, 6]. Genetic ablation of genes encoding SC components impedes meiosis and gametogenesis in mice [7], and mutation of these genes was also found in infertile patients [8]. Therefore, deciphering meiotic chromatin structures is valuable to demonstrate mechanistic steps for homologous chromosome recognition and recombination during meiosis. Despite intensive ultrastructural analysis [9–13], no attempts have been made to identify how chromatin sequences are organized by SYCP3 and related meiotic axial proteins [14].

Combinatorial interactions of epigenetic regulators remodel chromatin architecture to control meiosis, including the formation of chromosome axes, transcription regulation, DNA double-strand breaks (DSBs), meiotic sex chromosome inactivation, meiotic recombination, etc. [15]. In response to the alteration of epigenetic modifications [16, 17], transcriptional activities driven by RNA polymerase II (Pol II) are robust and dynamic at different stages in the meiotic prophase [18] and may be involved in homologous chromosome association [19]. Moreover, a programmed DSB event is a distinctive feature of meiosis [20, 21]. DSBs are marked by γH2A.X in eukaryotic cells, which is generated by serine phosphorylation of the histone H2A variant H2A.X and is essential for male meiosis and fertility [22]. Spo11 was reported to generate meiotic DSBs for synapsis and meiotic recombination [23, 24]. Asynapsed sex chromosomes with retained DSBs form XY body with inactivated chromosomes [25]. SYCP3 protein localization changes with chromosome morphology in autosomes and is involved in maintaining the association of sex chromosomes [26]. However, how epigenetic modifications at specific DNA sequences interact with SYCP3 and related meiotic axial proteins remains to be determined.

In this study, we aim to document genome-wide occupancy of SYCP3 in meiotic prophase and identify how its interaction with chromatin is regulated.

## Materials and methods

### Mice

Mice were maintained on C57BL/6J background. *Scml2* KO mice were as previously described [11]. Animal experiments were conducted under the approval of the Institutional Animal Care and Use Committee of Tongji Medical College, Huazhong University of Science and Technology. Animal experiments were conducted ethically according to the Guide for the Care and Use of Laboratory Animal guidelines.

### Separation of mouse spermatocytes

Separation of spermatocytes was performed as previously described [27]. Generally, testes were isolated from male mice at post-natal day 10 (P10; for leptotene spermatocytes), 4 weeks (for mixed spermatocytes), or adult (8 weeks or older; for pachytene spermatocytes). Collagenase (Sigma) and trypsin (Sigma) were used to remove interstitial cells and digest spermatogenic cells. Subsequently, a discontinuous bovine serum albumin (BSA, Sangon Biotech, China) density gradient was used to isolate spermatocytes at specific developmental stages.

### Modified CUT&Tag assay

CUT&Tag was performed according to the manufacturer’s protocol (Hyperactive *In Situ* ChIP Library Prep Kit for Illumina, Vazyme, China) with modifications. Antibodies against SYCP3 (sc-74568, mouse origin, Santa Cruz, USA; ab15093, rabbit origin, Abcam, USA), SYCP1 (ab15090, Abcam, USA), γH2A.X (ab26350, Abcam, USA), and RPB1 of Pol II (39497, Active Motif, USA) were used. Goat Anti-Mouse IgG H&L (ab6708, Abcam, USA) and goat Anti-Rabbit IgG H&L (ab6702, Abcam, USA) were used as negative controls. 2–5 × 10^4^ cells were used per reaction, and two to three replicates were performed for each condition. Generally, cells were immobilized with concanavalin A-coated magnetic beads and permeabilized by digitonin, followed by reaction at room temperature with primary antibody, corresponding secondary antibody, and pG-Tn5 transposase to cleave the genome adjacent to a target protein with adapters added at the same time. 1 U/μl RNaseOUT (10777019, Thermo Fisher, USA) was added to the reaction in the whole procedure. One microliter of DNase/RNase-free glycogen (AM9510, Thermo Fisher, USA) was added during DNA precipitation. Polymerase Chain Reaction (PCR) was performed to generate the library. Qualified libraries were then loaded on the Illumina Hiseq platform for PE150 sequencing.

### 1,6-hexanediol treatment of mouse spermatocytes

To obtain transiently permeabilized cells, spermatocytes were incubated with 5% digitonin in wash buffer (Dig-wash buffer, Vazyme, China) for 3 min on ice. Then spermatocytes were incubated with 5% 1,6-hexanediol (1,6-HD) (Sangon Biotech, China) in Dig-wash buffer for 1 h at room temperature before the CUT&Tag experiment.

### Expression of recombinant mouse SYCP3 protein *in vitro*

Recombinant mouse SYCP3 protein with 6× His tag was constructed by Absin (China). Generally, the plasmid containing T7 promoter and mouse *Sycp3* gene with C-terminal 6× His tag was constructed and introduced into *E. coli* for *in vitro* synthesis of SYCP3 protein, followed by protein extraction and purification. Mouse SYCP3 protein sequence used for recombinant protein expression is as following: MLRGCGDSDSSPEPLSKHLKMVPGGRKHSGKSGKPPLVDQPKKAFDFEKDDKDLSGSEEDVADEKAPVIDKHGKKRSAGIIEDVGGEVQNMLEKFGADINKALLAKRKRIEMYTKASFKASNQKIEQIWKTQQEEIQKLNNEYSQQFMNVLQQWELDIQKFEEQGEKLSNLFRQQQKIFQQSRIVQSQRMFAMKQIHEQFIKSLEDVEKNNDNLFTGTQSELKKEMAMLQKKVMMETQQQEMANVRKSLQSMLF.

### Electrophoretic mobility shift assay

The EMSA Probe Biotin Labeling Kit (GS008, Beyotime, China) was used to label single-stranded DNA (ssDNA) probes (60 nt). Biotin-labeled dUTP was added to the 3′ end of ssDNA by terminal deoxynucleotidyl transferase in a template-independent manner. The two ssDNA sequences of the probe were labeled separately and subsequently annealed to form double-stranded probe for subsequent electrophoretic mobility shift assay (EMSA) experiment. Unlabeled ssDNA sequences were annealed to form double-stranded competitors. Chemiluminescent EMSA Kit (GS008, Beyotime, China) was used to examine shifted biotin-labeled probes by streptavidin-HRP and BeyoECL Moon reagents. Generally, 15 μl of reaction systems were prepared by mixing Gel-Shift binding buffer, recombinant SYCP3 protein, labeled probe, and unlabeled competitors. The reaction system was incubated for 1 h at room temperature, followed by electrophoresis in 4% polyacrylamide gel for 45 min. Probe-protein complexes at polyacrylamide gel were transferred to nylon membranes on ice. The nylon membrane was then treated with a UV-light cross-linker for 15 min, followed by treatment with sealing solution at room temperature for 15 min, and then with streptavidin-HRP conjugate for 30 min. After three times with washing solution and treatment with equilibrium solution, fluorescence was enhanced using the BeyoECL Moon developer and examined (Shanghai JP Analytical Instrument Co., Ltd). The DNA probes used for EMSA were synthesized by Suzhou GentleGen Biotechnology (Suzhou, China); for methylation of DNA probes, all cytosines in the sequence of probe-5mC were 5-methyl-deoxycytosine and abbreviated as 5mC. Please see Supplementary Table S1 for EMSA probe sequences.

### Co-immunoprecipitation

Testes from wild-type male mice were homogenized in 1 ml lysis buffer (Beyotime, China) with a 1% Protease Inhibitor Cocktail (Beyotime, China). The lysates were incubated with an anti-SYCP3 antibody (sc-74568, Santa Cruz, USA) or anti-RPB1 antibody (39497, Active Motif, USA) as per the manufacturer’s protocol (P2179, Beyotime, China), followed by immunoblotting with either anti-SYCP3 or anti-RPB1 antibodies. Anti-RPB3 antibody (13428-1-AP, Proteintech, China) was also used for immunoblotting examination. To check potential impact of DNA/RNA contamination on co-immunoprecipitation result, 5 μg/l DNase I (A610099, Sangon Biotech, China) or RNase A (EN0531, Thermo Scientific, USA) was added to testicular lysate for 30 min at room temperature, followed by incubation of the reaction with antibodies.

### Spermatocyte culture and treatment

Pachytene spermatocytes were obtained by a discontinuous BSA density gradient. The cell pellet was resuspended in culture medium Minimum Essential Medium (MEM) with 10% Fetal bovine serum (FBS) at a concentration of 10^5^ cells/ml and cultured at 37°C in a 5% CO_2_ incubator. To examine the impact of transcription activity on meiotic chromatin structure, pachytene spermatocytes were cultured with or without 20 μM α-amanitin (MedChemExpress, USA) for 12 h. To examine the impact of histone acetylation on meiotic chromatin structure, pachytene spermatocytes were cultured in culture medium with 100 nM Trichostatin A (TSA) (Merck, Germany) for 3 h, followed by TSA washout and recovery in culture medium without TSA for another 3 h. Spermatocytes with or without drug treatment were collected at indicated time points for further examination.

### Chromosome spreads and immunofluorescence

Spermatocyte chromosome spreads were performed as described [28]. Spermatocytes were de-capsulated into 100 mM sucrose. Next, cells were added to slides covered with 1% paraformaldehyde and then dried in a humidified chamber. Slides were washed with 0.4% Photo-Flo 200 solution and dried for immunofluorescence. Spermatocyte chromosome spreads were blocked with 1% BSA in Phosphate Buffered Saline (PBS) for 1 h at room temperature and then incubated with primary antibody overnight at 4°C. The following primary antibodies were used for immunostaining on chromosome spread, SYCP3 (sc-74568, Santa Cruz, USA; ab15093, Abcam, USA), γH2A.X (ab26350, Abcam, USA), RPB1 of Pol II (39497, Active Motif, USA), and REC8 (HPA031727, Sigma, USA). After incubation with Alexa Fluor secondary antibodies, samples were examined under a confocal microscope (Zeiss).

### Data analysis of WGBS data

Publicly available whole genome bisulfite sequencing (WGBS) data of mouse spermatocytes was downloaded from GSM2674824 for data processing. Raw reads were processed with Trim Galore (v0.6.4) to remove adaptor sequences and poor-quality bases with “–q 20 –phred33 –stringency 5 –length 20 –paired.” Trimmed reads were then aligned to the mouse reference genome (mm10) using Bismark (v0.22.3) with default parameters. SAMtools (v1.3.1) was used to sort bam files by genomic coordination and make a bam file index. PCR duplicates were removed using Picard (v2.26.6). The methylation ratio at each CpG site was constructed using the bismark_methylation_extractor model with the parameters “-p -comprehensive -no_overlap -bedgraph -counts -report -cytosine_report -gzip -buffer -size 30G.” The UCSC genome browser utility, bedGraphToBigWig, was used to transform the bedgraph files into bigwig files. UCSC genome browser was used for visualization. The methylation levels at CpG sites were first calculated by the “methRead” function of R package methylKit (v1.14.2) with mincov = 3. Methylation across the genome was tiled with the “tileMethylCounts” function using the parameters “win.size = 1000, step. size = 1000,” then the “unite” function was used to unite tiled regions with the “destrand = TRUE” parameter. CpG sites with methylation levels <25% were defined as hypomethylation sites, CpG sites with methylation levels above 75% were defined as hypermethylation sites, and CpG sites with methylation levels between 25% and 75% were defined as intermediate methylation.

### CUT&Tag data analysis

Raw reads were processed with Trim Galore (v0.6.4) to remove adaptor sequences and poor-quality bases with “–q 20 –phred33 –stringency 5 –length 20 –paired.” Trimmed reads were then aligned to the mouse reference genome (mm10) using Bowtie2 (version 2.4.1) with default parameters. Picard (v2.26.6) was used to remove PCR duplicates. CUT&Tag data were normalized to reads per kilobase per million mapped reads. Correlation of different samples was calculated by multiBamSummary/multiBigwigSummary and plotCorrelation of the Python package deepTools (v3.5.1). Principal components analysis (PCA) was performed by R package DiffBind (v3.10.1). Density plots of signal enrichment were generated by bamCoverage, computeMatrix, and plotHeatmap of the Python package deepTools (v3.5.1). Genomic coordinates for individual features, including Transcription Start Site (TSS), Untranslated Regions (UTRs), Coding Sequence (CDS), and introns were obtained from the UCSC genome browser. Peaks were called by MACS2 (v2.2.7.1) with default parameters. R package circlize was used to depict peak distribution along chromosomes. Peak annotation was performed by R package ChIPseeker (v1.34.1) using genomic coordinates downloaded from the UCSC genome browser for mouse genome or repeat elements. GC content for peaks was calculated by map of bedtools utilities (v2.29.2). Peaks with a distance of <1 kb were combined using the R package GenomicRanges (v1.40.0). Motif analysis was performed by HOMER (v4.11.1), using mouse genome as background DNA sequence. The width of peaks was obtained by subtracting the start coordinates from the end coordinates of identified peaks. The peak distance was calculated by bedtools closest (v2.29.2). Venn diagrams of peak overlapping were plotted by findOverlapsOfPeaks of R package ChIPpeakAnno (v3.34.1). To obtain SYCP3-binding genic regions, SYCP3 peaks were annotated, and genes with their promoter/UTRs/exon/intron/downstream regions bound by SYCP3 were used for further analysis. The enrichment of transposable elements (TEs) was calculated by the ratio of the length of TEs in peaks to the width of total peaks versus the ratio of the length of TEs to the chromosome size of the mouse genome. For the heatmap of specific histone modifications enriched at individual TE families, we first calculated normalized counts per TE transcripts by multiplying the density of individual TE transcript by its length, then we summarized counts of all TE transcripts belonging to the specific TE family, and finally we divided these counts by the total length of the specific TE family. Violin plot, pie plot, heatmap, histogram, dot chart, and box plot were generated by ggplot2 in R (v3.3.2). CUT&Tag dataset generated in this study was in Supplementary Table S2. MACS2-called peaks were summarized in Supplementary Table S3. Occupancy of SYCP1 and SYCP3 at repetitive elements was summarized in Supplementary Table S4. Gene information for SYCP3-Core sites was in Supplementary Table S5.

### Statistical analyses

Statistical tests were performed using R (v4.0.2). A significant difference between different pooled samples was determined using the Wilcoxon rank-sum test. *P* < .05 was considered to be statistically significant.

## Results

### SYCP3 protein is enriched at open chromatin regions during meiosis

To map genome-wide occupancy of SYCP3 in mouse spermatocytes, we collected spermatocytes from 4-week-old male mice and performed modified CUT&Tag using two different anti-SYCP3 antibodies of mouse and rabbit origin, respectively. The peak distribution using the two antibodies (Supplementary Fig. S1A) was highly correlative (Supplementary Fig. S1B). In contrast, there were almost no identified peaks when NIH3T3 cells were used in the CUT&Tag experiment (Supplementary Fig. S1C). These results document non-random SYCP3 occupancy at meiotic chromatin, and mouse anti-SYCP3 antibody was used for further experiments.

**Figure 1.**
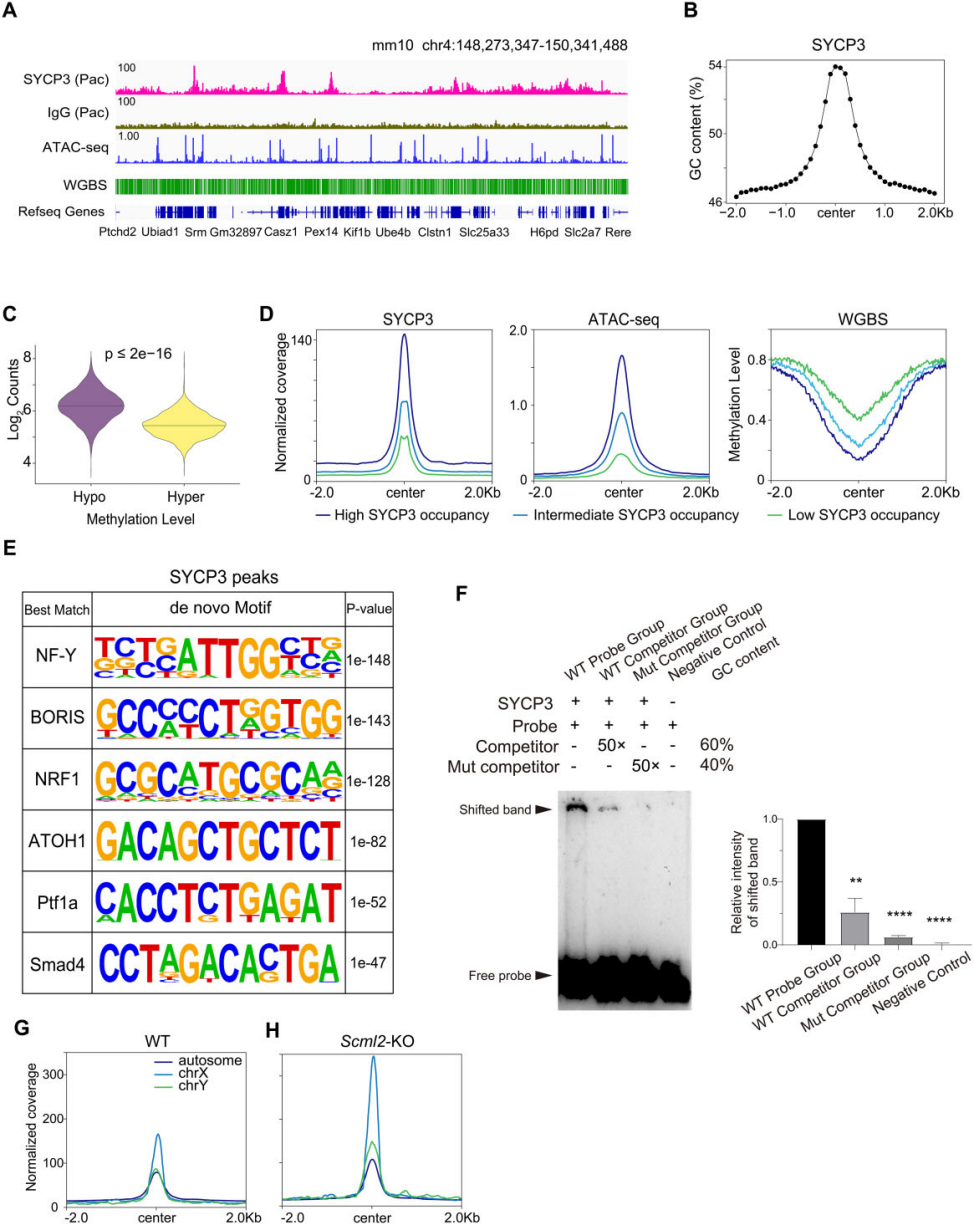
SYCP3 occupancy in meiotic chromatin identified by modified CUT&Tag. (**A**) Integrative Genomics Viewer (IGV) genome browser snapshots of SYCP3 occupancy in pachytene spermatocytes with IgG as negative control, ATAC-seq of spermatocytes, and WGBS signals of spermatocytes. (**B**) Density plot of GC content around the peak center of SYCP3 (±2 kb) in pachytene spermatocytes. Each dot represents mean GC content using a 100 bp-wide rolling window. (**C**) Violin plots of log_2_(normalized counts) of SYCP3 enrichment in hypomethylation and hypermethylation regions in spermatocytes. *P* value was calculated by the Wilcoxon rank-sum test. (**D**) Read density plots of the distribution of SYCP3 peaks, ATAC-seq, and WGBS signals at high/intermediate/low SYCP3 occupancy regions (center ± 2.0 kb) in spermatocytes. (**E**) Putative motifs strongly enriched in SYCP3-binding sequences in pachytene spermatocytes. (**F**) EMSA assay showing the binding activity of recombinant SYCP3 protein to the double-stranded DNA (dsDNA) probe. 0.2 μM biotin-labeled probe and 1 μg of SYCP3 protein were added per reaction. Probe sequence was selected based on positive SYCP3 signal at meiotic chromatin in mouse spermatocytes. Mutated (Mut) sequence was obtained through G/C-to-A/T and A/T-to-G/C substitution of WT sequence. Probe/competitor sequence, forward: 5′-ACAGTCCTGGTGATTGAACTCCGGCCCTGGGCATGCCAGGCGAGCACTCCACTCTTTGAG-3′; reverse:5′-CTCAAAGAGTGGAGTGCTCGCCTGGCATGCCCAGGGCCGGAGTTCAATCACCAGGACTGT-3′. Mut competitor sequence, forward: 5′-GTGACTTCAACAGCCAGGTCTTAATTTCAAATGCATTGAATAGATGTCTTGTCTCCCAGA-3′; reverse: 5′-TCTGGGAGACAAGACATCTATTCAATGCATTTGAAATTAAGACCTGGCTGTTGAAGTCAC-3′. For competition assays, a 50-fold excess of unlabeled probe was added. GC contents of the sequences were labeled as indicated. Bar graph showing the ratio of the gray value of samples from different lanes to that of the WT probe group. *n* = 3 per group. *P* value was calculated by the Student’s *t*-test. Enrichment of SYCP3 on autosomes and sex chromosomes in WT (**G**) and *Scml2*-KO (**H**) spermatocytes (center ± 2.0 kb).

To characterize SYCP3-enriched chromatin regions, we performed CUT&Tag using purified pachytene spermatocytes in which stable SC structures are formed to hold homologous chromosomes together (Fig. 1A and Supplementary Fig. S1D). Sequence analysis of SYCP3 peaks revealed high GC content in proximity of peak centers (Fig. 1B), similar to Pol II (Supplementary Fig. S1E). Further analysis using WGBS of mouse spermatocytes showed that SYCP3 was associated with DNA hypomethylation regions than intermediate/hypermethylation regions (Supplementary Fig. S1F). DNA hypomethylation regions had higher SYCP3 occupancy (Fig. 1C and Supplementary Fig. S1G). Next, we ordered SYCP3-occupied sequences into three groups based on SYCP3 enrichment. We found that stronger SYCP3 occupancy was correlated with higher chromatin accessibility by ATAC-seq and lower DNA methylation by WGBS (Fig. 1D), indicating that DNA sequence and chromatin modifications are involved in SYCP3 association with chromatin [29]. Notably, the HOMER algorithm [30] of SYCP3 peaks identified enriched motifs of several transcription factors, including NF-Y, Brother of the regulator of the imprinted sites (BORIS), and NRF1 (Fig. 1E). NF-Y is a highly conserved transcription factor that maintains high transcriptional fidelity and acts as a promoter organizer during spermatogenesis [31, 32]. Expression of BORIS is normally restricted to male germ cells, and the combination of BORIS and CCCTC binding factor (CTCF) ensures normal spermatogenesis by repressing pre-meiotic genes and activating post-meiotic genes [33]. NRF1 has been shown to regulate mitochondrial biogenesis, and ablation of NRF1 led to abnormal male germ cell gene expression [34].

It was reported that SYCP3 protein directly interacts with dsDNA [3], so we ask whether recombinant SYCP3 has sequence-specific recognition of DNA target sites. Here, we purified recombinant His-tagged full-length mouse SYCP3 protein, followed by sodium dodecyl sulfate–polyacrylamide gel electrophoresis (SDS–PAGE) (Supplementary Fig. S1H) and antibody verification (Supplementary Fig. S1I). Then we performed an EMSA assay to examine *in vitro* DNA-binding activity of SYCP3, using the probe sequence derived from SYCP3 CUT&Tag peak summit in pachytene spermatocyte. Our result showed that the recombinant SYCP3 protein exhibited binding capability to dsDNA probes, and this binding was efficiently competed by unlabeled competitor with the same sequence (Fig. 1F). The competition assay further revealed that mutated sequence with G/C-to-A/T and A/T-to-G/C substitution, which led to decreased GC content remained associated with SYCP3 with higher binding affinity (Fig. 1F). This finding was corroborated by subsequent EMSA experiment using both unmutated and mutated probe sequences, with consistent results observed for another probe sequence derived from SYCP3 CUT&Tag peaks (Supplementary Fig. S1J). To further characterize the features of SYCP3-DNA interaction, we used ssDNA sequences as probes and observed significant reduction of shifted bands (Supplementary Fig. S1K), indicating that SYCP3 mainly binds to dsDNA molecules. Since protein–DNA interaction *in vivo* is also affected by epigenetic modifications, we focused on the impact of DNA methylation on DNA-binding ability of recombinant SYCP3. EMSA assay demonstrated that methylation of the dsDNA probe significantly weakened SYCP3-DNA-binding affinity (Supplementary Fig. S1L), suggesting that DNA methylation modification negatively affects SYCP3-DNA interaction. Additionally, increase of NaCl concentration to 2.5M in the reaction system significantly hindered binding activity of SYCP3 (Supplementary Fig. S1M). Therefore, our findings demonstrate that recombinant SYCP3 exhibits higher binding affinity to dsDNA with lower GC content *in vitro*, whereas ssDNA and methylation-modified dsDNA bind weakly to recombinant SYCP3 and, furthermore, high NaCl concentration eliminates DNA binding of recombinant SYCP3.

Further analysis documented that SYCP3 is associated with both autosomes and sex chromosomes in pachytene spermatocytes (Fig. 1G). Sex chromosomes are decorated with sex comb on midleg-like 2 (SCML2), a germ cell-specific chromatin-binding protein that antagonized H2A monoubiquitination in male meiosis [11, 35]. To identify how *Scml2* deficiency impacts chromatin binding activity of SYCP3, we profiled genome-wide SYCP3 occupancy in pachytene spermatocytes from *Scml2* KO mice (Fig. 1H). We found that SYCP3 enrichment at sex chromosomes, especially X chromosome, was significantly enhanced. Notably, it has been shown that the transcription activity of X chromosome was mildly derepressed in *Scml2*-deficient pachytene spermatocytes [35]. Therefore, our results demonstrate that SCML2, which counteracts H2A monoubiquitination, inhibits SYCP3 occupancy at sex chromosomes, especially X chromosome.

Meiotic chromosome axis structure hints at potential periodicity of SYCP3 association with chromatin. Therefore, we calculated the distribution of distances between adjacent SYCP3 peaks. We found that SYCP3 peak distances ranged from 1 to 10 kb (Supplementary Fig. S1N), and the density plot showed no periodicity of SYCP3 peaks in the mouse genome (Supplementary Fig. S1O).

Taken together, our results show that SYCP3 mainly occupies meiotic chromatin regions that have high GC content, low DNA methylation, and high chromatin accessibility in mouse spermatocytes.

### SYCP3 occupancy is highly correlated with Pol II enrichment at meiotic chromatin

**Figure 2.**
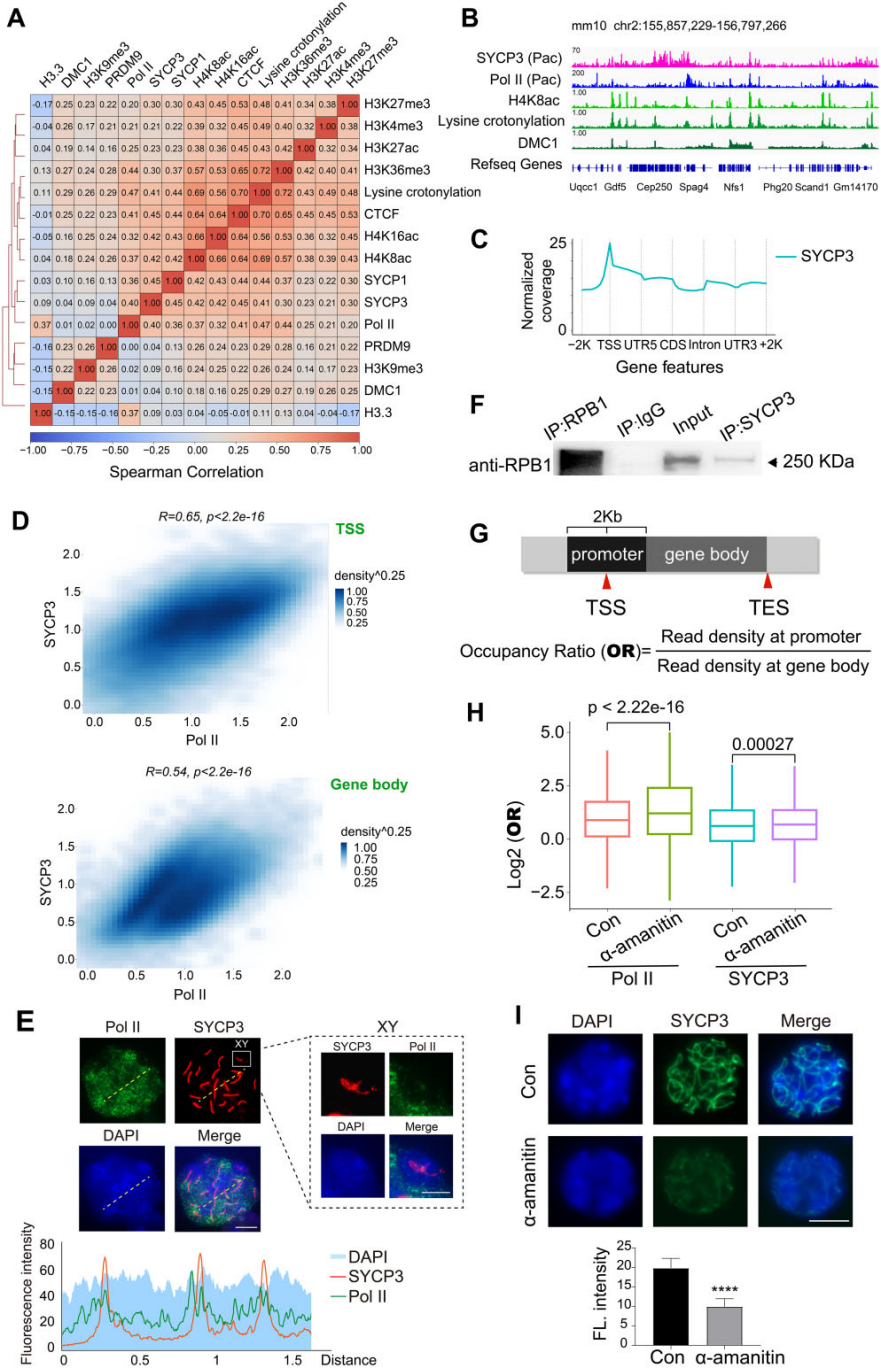
SYCP3 occupancy is positively correlated with active chromatin marks and Pol II enrichment in spermatocytes. (**A**) Heatmap of pairwise Spearman correlation of chromatin association of SYCP3/1, histone marks, Pol II, CTCF, and DMC1-labeled DSB hotspots in spermatocytes. (**B**) IGV genome browser snapshots of enrichment of SYCP3, Pol II, H4K8ac, lysine crotonylation, and DMC1 in spermatocytes. (**C**) Diagram showing SYCP3 distribution along genic regions in spermatocytes. (**D**) The 2D density plots of SYCP3 intensity versus Pol II intensity at gene promoters/gene body regions show a positive correlation. Here, TSS regions were defined as 1 kb flanking TSS. Gene body regions were defined as 1 kb downstream of TSS to Transcription End Site (TES). (**E**) Co-immunostaining of SYCP3 and Pol II in meiotic chromosome spread of pachytene spermatocytes. DNA was stained by 4',6-diamidino-2-phenylindole (DAPI). Scale bar, 10 μm. The line chart shows the distribution of the fluorescence intensities along the dashed line (lower). Immunofluorescence of the sex chromosome portion is shown on the right. Scale bar, 5 μm. (**F**) Immunoblotting of RPB1 (the largest subunit of Pol II) after immunoprecipitation with IgG, anti-RPB1 antibody, and anti-SYCP3 antibody from adult testicular lysate, using input without immunoprecipitation as positive control. (**G**) Scheme of occupancy ratio (OR) definition as chromatin occupancy (quantified as normalized read density) at the promoter (1 kb flanking TSS) versus gene body (1 kb downstream of TSS to TES). (**H**) Boxplot indicating changes of OR values of Pol II and SYCP3 occupancy upon α-amanitin treatment. *P* value was calculated by the Wilcoxon rank-sum test. (**I**) Immunofluorescence staining and the fluorescence intensity of SYCP3 in chromosome spread of pachytene spermatocytes from control and α-amanitin treatment group. Scale bar, 10 μm.

To investigate epigenetic regulation of SYCP3 occupancy at meiotic chromatin, correlation analysis was performed among profiles of SYCP3 and other epigenetic marks, including histone modifications [36–39], Pol II, CTCF [40], and DMC1-labeled DSB hotspots [41] (Fig. 2A and B). As expected, SYCP3 occupancy was positively correlated with active chromatin marks like H4K8ac and lysine crotonylation, and had much weaker correlation with heterochromatin mark H3K9me3. Notably, the SYCP3 profile had a strong correlation with Pol II, indicating that SYCP3 may largely associate with chromatin regions opened by Pol II progression during transcription. Interestingly, we noticed that SYCP3 and CTCF occupancy were also highly correlated. Due to the interaction and cooperation of CTCF and Pol II [42], positive roles of CTCF on transcription seemed to be involved in organizing the meiotic chromosome axis.

To decipher the relationship between SYCP3 occupancy and transcription event, we profiled SYCP3 occupancy at genic regions and found that SYCP3 was indeed enriched in the proximity of the promoter and gene body (Fig. 2C). In agreement with this result, both SYCP3 and Pol II peaks were abundant at genic regions (Supplementary Fig. S2A and B), and stronger SYCP3 occupancy was correlated with stronger Pol II occupancy (Supplementary Fig. S2C). Moreover, positive correlation was identified between intensities of SYCP3 and Pol II at both promoter and gene body regions (Fig. 2D). Interestingly, we noticed that immunofluorescence signals of SYCP3 and Pol II had significant overlap in meiotic chromosome spread (Fig. 2E), and co-immunoprecipitation assay demonstrated interaction between SYCP3 and RPB1, the largest subunit of Pol II (Fig. 2F and Supplementary Fig. S2D). Interaction between SYCP3 and Pol II was also supported by co-immunoprecipitation assay of SYCP3 and RPB3, another Pol II subunit (Supplementary Fig. S2E). To prevent impact of potentially contaminated DNA/RNA on co-immunoprecipitation result, we cultured cell lysate with DNase/RNase before immunoprecipitation of protein complexes and confirmed SYCP3-RPB3 interaction in the presence of DNase/RNase (Supplementary Fig. S2F).

Then we asked whether transcription activity impacts SYCP3 occupancy. We treated freshly purified pachytene spermatocytes with α-amanitin, which blocks transcription elongation. We then examined the chromatin binding of Pol II and SYCP3 by CUT&Tag (Supplementary Fig. S2G), focusing on genic regions with MACS2-called SYCP3 peaks. As expected, at SYCP3-associated genic regions, we identified reduced Pol II signal in gene bodies and arrested Pol II at promoter after α-amanitin treatment (Supplementary Fig. S2H). Distribution pattern of SYCP3 at genic regions was similar upon α-amanitin treatment (Supplementary Fig. S2I), and SYCP3 enrichment at promoter regions was not affected (Supplementary Fig. S2J). We then define OR as read density at promoters versus read density at gene bodies to quantify protein occupancy at promoters relative to gene bodies (Fig. 2G). We observed a significantly increased OR value of Pol II and a mildly increased OR value of SYCP3 upon transcription elongation inhibition (Fig. 2H). In agreement with this result, our immunofluorescence result showed reduced SYCP3 intensity at chromatin upon α-amanitin treatment (Fig. 2I). These results suggest that transcription activation facilitates SYCP3 association at meiotic chromatin.

Taken together, our results show that SYCP3 occupancy at meiotic chromatin is dynamic and positively correlated with active chromatin marks and transcription by Pol II.

### SYCP3 occupancy is gradually accumulated in the progression of the meiotic prophase

**Figure 3.**
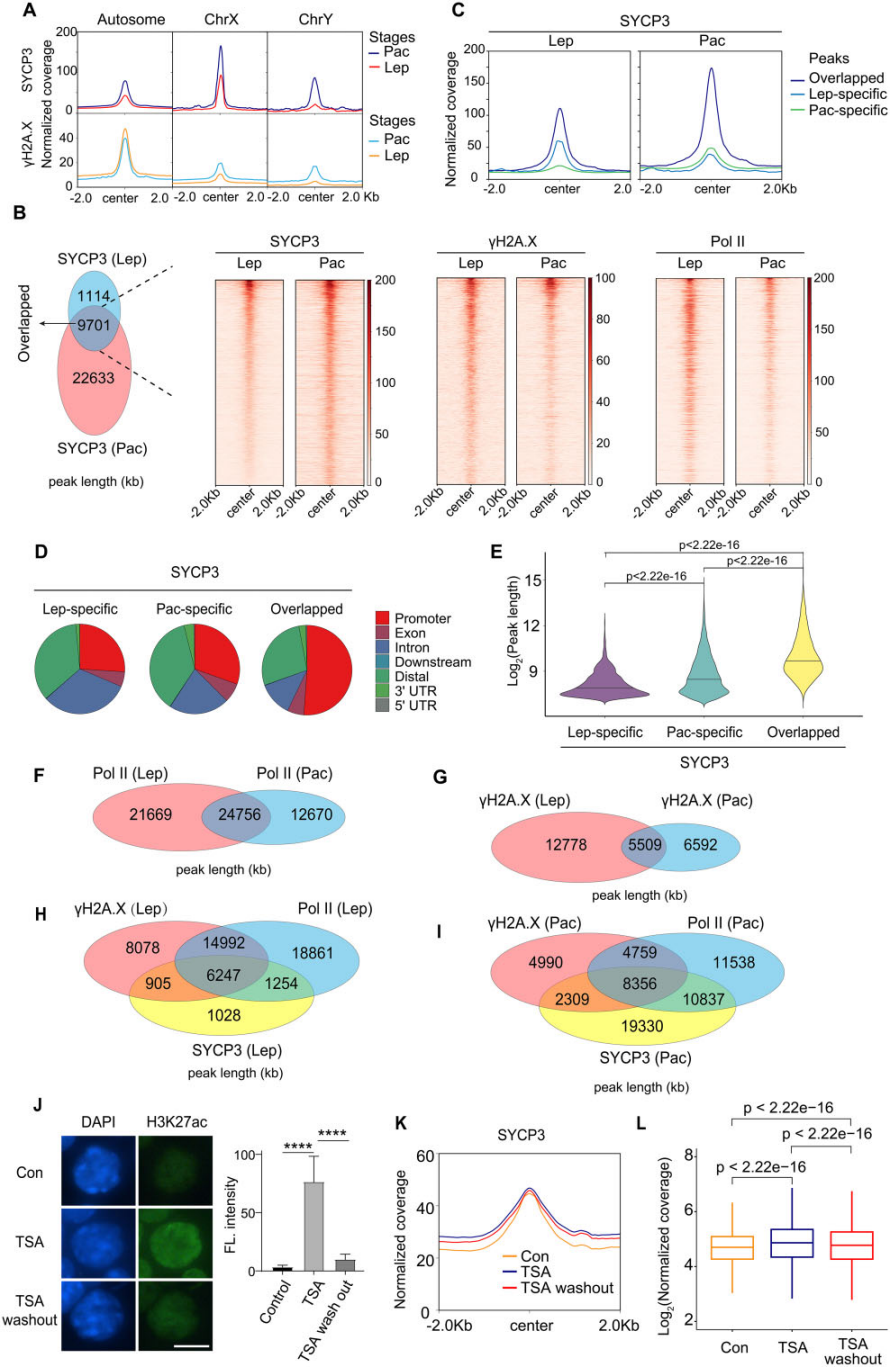
Comparison of chromatin occupancy of SYCP3, Pol II, and γH2A.X in leptotene and pachytene spermatocytes. (**A**) Changes of the enrichment of SYCP3 and γH2A.X on the autosome, X and Y chromosome in leptotene (Lep) and pachytene (Pac) spermatocytes (2.0 kb flanking peak center). (**B**) Venn diagram showing the overlap of SYCP3 peaks in pachytene and leptotene spermatocytes. Heatmap showing the enrichment of SYCP3, γH2A.X, and Pol II in leptotene and pachytene spermatocytes at overlapped peak regions of the two stages. (**C**) Density plot of SYCP3 occupancy at leptotene-specific, pachytene-specific, and overlapped SYCP3 peaks (2.0 kb flanking peak center) in leptotene and pachytene spermatocytes. (**D**) Pie plots indicating genomic distribution of SYCP3 binding at stage-specific peaks and overlapping peaks in leptotene and pachytene spermatocytes. (**E**) Peak length distribution of stage-specific and overlapping SYCP3 peaks in leptotene and pachytene spermatocytes. (**F**, **G**) Venn diagrams showing the overlap of Pol II/γH2A.X peaks in pachytene and leptotene spermatocytes. (**H**, **I**) Venn diagrams showing the overlap of SYCP3, Pol II, and γH2A.X peaks in pachytene and leptotene spermatocytes. (**J**) Immunofluorescence staining and the fluorescence intensity of H3K27ac in pachytene spermatocytes. DNA was stained by DAPI. Con group, control without drug treatment. TSA group, pachytene spermatocytes were treated with 100 nM TSA for 3 h. TSA washout group, pachytene spermatocytes were recovered for 3 h without drugs after TSA treatment. (**K**) Intensity of SYCP3 occupancy at SYCP3-binding sites in Con, TSA, and TSA washout groups of pachytene spermatocytes (2 kb flanking peak center). (**L**) Boxplot shows quantification of SYCP3 occupancy at SYCP3-binding sites in Con, TSA, and TSA washout groups of pachytene spermatocytes. *P* value was calculated by the Wilcoxon rank-sum test.

Attachment of SYCP3 to meiotic chromatin has been visualized from as early as the leptotene stage of meiotic prophase I, when chromosomes start to condense and form threadlike structures. To examine SYCP3 occupancy at this stage, we purified leptotene spermatocytes from post-natal day 10 (P10) testes, where spermatocytes are mainly at the leptotene stage [43]. Immunofluorescence staining of meiotic chromosome spread using anti-γH2A.X and anti-SYCP3 antibodies confirmed the purity of collected leptotene spermatocytes from P10 testes (Supplementary Fig. S3A), which differed morphologically from pachytene spermatocytes in adult testes (Supplementary Fig. S1D). Then we compared SYCP3 occupancy in purified leptotene and pachytene spermatocytes (Supplementary Figs S2A and S3B). To obtain a global view of SYCP3 binding activity at the two stages, we combined MACS2-called SYCP3 peaks of the two stages and profiled SYCP3 distribution at the combined peak regions. We found that SYCP3 occupancy was greatly enhanced at both autosomes and sex chromosomes in pachytene compared to the leptotene stage (Fig. 3A), in agreement with the gradual accumulation of SYCP3 on chromatin during meiotic progression. To further explicate the transition of SYCP3 occupancy from leptotene to the pachytene stage, we compared identified SYCP3 peaks at the two stages (Fig. 3B). Notably, we found that SYCP3-occupied genomic regions at the leptotene stage were largely retained at the pachytene stage. We then profiled SYCP3 binding at stage-specific peaks and overlapping peaks of the two stages, which occupied promoter of genes with different biological functions (Supplementary Fig. S3C). We observed that SYCP3 occupancy at overlapping peaks was not only retained but significantly enhanced at the pachytene stage (Fig. 3C). Moreover, these overlapping peaks were more localized at promoter regions (Fig. 3D) and were relatively broader in width (Fig. 3E). Meanwhile, leptotene-specific SYCP3 peaks demonstrated higher Pol II association at leptotene stage, and pachytene-specific SYCP3 peaks demonstrated higher Pol II association at pachytene stage (Supplementary Fig. S3D).

To determine whether SYCP3 occupancy at the leptotene stage is correlated with Pol II, we examined the chromatin association of Pol II in leptotene spermatocytes (Supplementary Figs S2A and S3B). We noticed that Pol II peaks in leptotene and pachytene stages were quite different from each other (Fig. 3F). Intriguingly, a significant amount of SYCP3-occupied genomic regions in leptotene stage (∼80%) was identified in Pol II-occupied genomic regions at leptotene stage (Supplementary Fig. S3E). However, more epigenetic modifiers should be investigated to determine causality of SYCP3 occupancy since most of Pol II peaks in leptotene stage were not SYCP3 peaks.

DSBs, labeled by γH2A.X, play important roles in organizing the architecture of the meiotic chromosomes. We therefore performed CUT&Tag to examine the distribution of γH2A.X in leptotene and pachytene spermatocytes (Supplementary Figs S2A and S3B). We combined peaks at the two stages to profile γH2A.X occupancy changes, and found that its enrichment at sex chromosomes was greatly enhanced in pachytene stage when XY body is formed (Fig. 3A), confirming the γH2A.X occupancy data. We noticed that γH2A.X peaks in leptotene and pachytene stages were quite different from each other (Fig. 3G). Remarkably, SYCP3-occupied genomic regions in leptotene stage were largely identified in overlapping peaks of Pol II and γH2A.X at the same meiotic stage (Fig. 3H). This suggests that collaborative transcription and DSB events initiated SYCP3 attachment to meiotic chromatin at the leptotene stage. In contrast, similar results were not observed at the pachytene stage (Fig. 3I). Therefore, we propose that SYCP3 peaks gradually accumulate during meiotic progression, although some SYCP3 peaks may be formed *de novo* in each meiotic stage.

Then we asked whether the overlapping SYCP3 peaks of leptotene and pachytene stages were due to retained SYCP3 occupancy during meiotic progression or Pol II/γH2A.X enrichment in meiotic chromatin. We profiled SYCP3, γH2A.X, and Pol II at the overlapping SYCP3 peaks in the two stages and found that intensities of γH2A.X and Pol II significantly decreased in the pachytene stage. In contrast, the enhanced SYCP3 intensity supports the retention of SYCP3 during meiotic progression (Fig. 3B).

Next, we evaluated the occupancy of SYCP3, γH2A.X, and Pol II at SYCP3/γH2A.X/Pol II-specific or combinatorial peaks in leptotene and pachytene stages and found that the enrichment of SYCP3, γH2A.X, and Pol II at overlapping peaks of SYCP3&γH2A.X&Pol II were highest in both leptotene and pachytene stages (Supplementary Fig. S3F). This indicates that combined transcription and DSB events in the pachytene stage may facilitate strong SYCP3 attachment to chromatin.

DMC1 recombinase enrichment by single-strand DNA (ssDNA) ChIP-seq (DMC1–SSDS) has been used to map DSB hotspots [44, 41]. We examined SYCP3 occupancy at these DMC1-enriched regions and noticed a triple peak pattern of SYCP3 at these sites in leptotene/zygotene and pachytene stages (Supplementary Fig. S3G). This result shows that sequences flanking resected DNA ends associate with axis proteins after DSB formation and is in agreement with the notion that meiotic DSB repair occurs in the context of chromosome axis structure.

To elucidate the regulatory pattern of SYCP3 accumulation at meiotic chromatin, we also treated freshly purified pachytene spermatocytes with Trichostatin A (TSA, a well-characterized histone deacetylase inhibitor) for 3 h, followed by washout of TSA and recovery for another 3 h. As expected, we observed increased histone acetylation of spermatocytes after TSA treatment and reduction of histone acetylation after recovery upon TSA washout (Fig. 3J). Interestingly, our CUT&Tag examination (Supplementary Fig. S3H) showed that global SYCP3 occupancy was enhanced upon TSA treatment and SYCP3 occupancy was partially retained even upon TSA washout (Fig. 3K and L). Similarly, genic regions with SYCP3 peaks demonstrated clearly retained SYCP3 occupancy (Supplementary Fig. S3I and J).

Taken together, these results are consistent with that the *de novo* establishment of SYCP3 occupancy is facilitated by transcription, DSB, and other modifications of chromatin. Our findings suggest that the SYCP3 occupancy at meiotic chromatin is likely to be determined by both massive SYCP3 occupancy retained during meiotic progression and *de novo* formation of SYCP3 binding sites by Pol II and other epigenetic modifiers.

### Identification of core SYCP3 binding sites with SYCP3 occupancy maintained upon 1,6-hexanediol treatment

**Figure 4.**
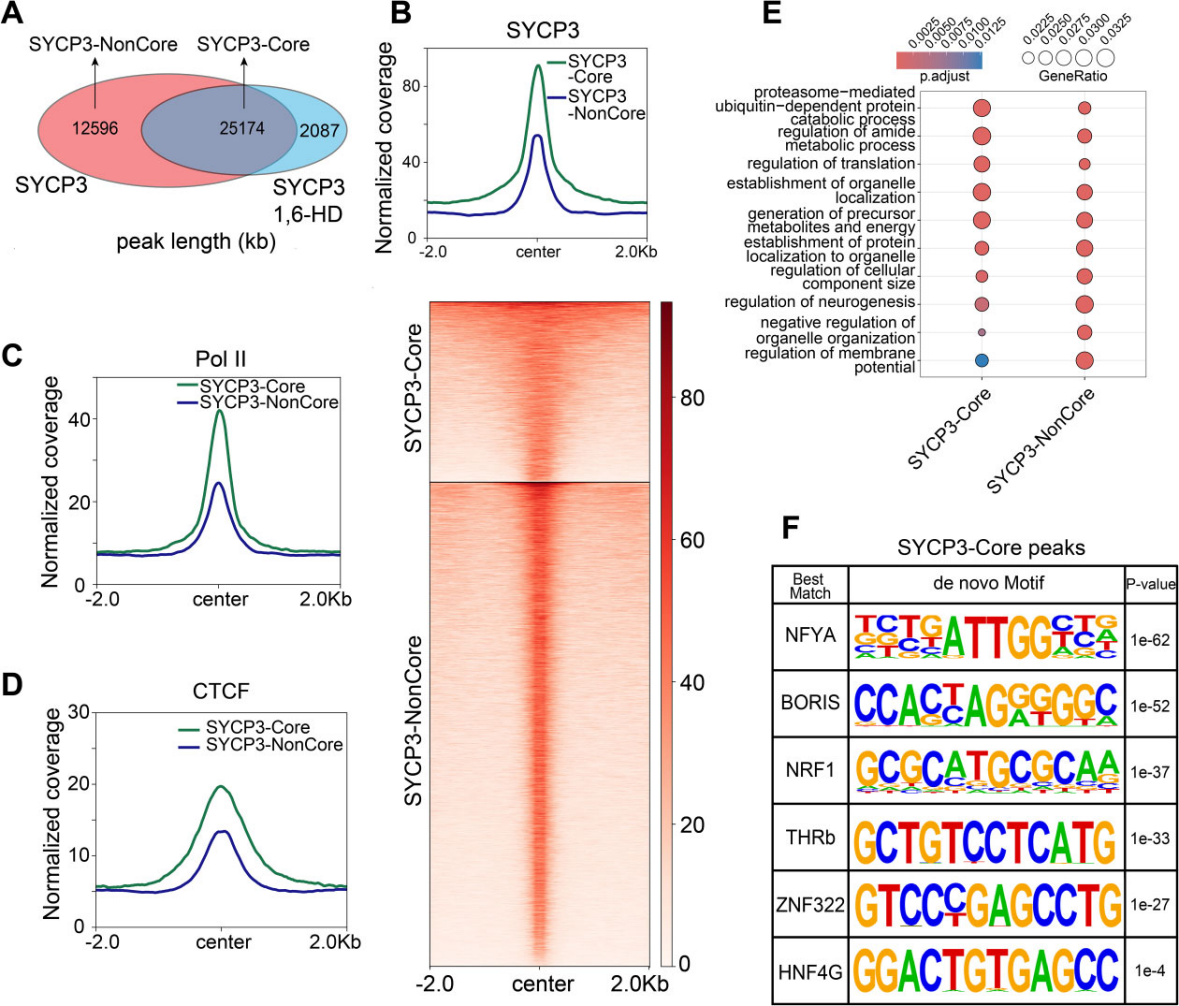
Identification and characterization of core SYCP3 binding sites. (**A**) Venn diagram showing the overlap of SYCP3 peaks in pachytene spermatocytes without (SYCP3) and with 1,6-HD treatment (SYCP3-1,6-HD). (**B**) Intensity and heatmap of SYCP3 occupancy at SYCP3-Core and SYCP3-NonCore sites in pachytene spermatocytes (2 kb flanking center). (**C**, **D**) Intensity of Pol II and CTCF occupancy at SYCP3-Core and SYCP3-NonCore sites in pachytene spermatocytes (2 kb flanking center). (**E**) Gene Ontology analysis of genes with promoters containing SYCP3-Core sites or SYCP3-NonCore sites. (**F**) Putative binding motifs enriched in SYCP3-Core sites by HOMER algorithm.

During meiosis, SYCP3 forms fibrous assemblies in spermatocytes for chromosome axis assembly [3, 45]. We then ask whether SYCP3 fibrous assembly formation facilitates SYCP3 attachment to meiotic chromatin. Generally, immunostaining of chromosome spread for SYCP3 showed inefficient SYCP3 fibrous assembly formation upon treatment with 5% aliphatic alcohol 1,6-HD, which disrupts explicitly weak hydrophobic interactions and impairs phase-separated structures [46] (Supplementary Fig. S4A). Additionally, staining of REC8, another important component of meiotic chromosome axis, demonstrated that chromosome axis structure was partially impaired upon 1,6-HD treatment (Supplementary Fig. S4A). Further examination of SYCP3 occupancy (Supplementary Fig. S4B) showed that ∼67% of SYCP3-occupied regions were retained upon 1,6-HD treatment (Fig. 4A). At these retained SYCP3 peak regions, SYCP3 occupancy was also reduced upon 1,6-HD treatment (Supplementary Fig. S4C). In agreement with CUT&Tag result, EMSA was performed to show modest reduction of DNA-binding ability of recombinant SYCP3 upon 1,6-HD treatment (Supplementary Fig. S4D).

Notably, retained SYCP3 peaks upon 1,6-HD treatment demonstrated stronger SYCP3 binding and were broader than the rest of the peaks (Fig. 4B). We conclude that these retained peaks are core attachment sites with stable SYCP3 binding, and the rest of the peaks seem to be indirectly formed with facilitation of SYCP3 fibrous assemblies. We named the retained peaks that persist after 1,6-HD treatment and the rest of the peaks as “SYCP3-Core” and “SYCP3-NonCore” sites, respectively. Moreover, SYCP3-Core sites demonstrated stronger Pol II and CTCF occupancy than SYCP3-NonCore sites (Fig. 4C and D). Gene Ontology analysis indicated that specific biological processes such as proteostasis and organelle localization are enriched in genes with promoters containing SYCP3-Core sites, relative to SYCP3-NonCore sites (Fig. 4E). Using the HOMER algorithm on the SYCP3-Core peaks, we identified enriched motifs for transcription factors including NFYA, BORIS, and ZNF322 (Fig. 4F).

Taken together, we identified core SYCP3-binding sites in pachytene spermatocytes, which were largely resistant to 1,6-HD treatment to dissolve SYCP3 fibrous assemblies. These core binding sites have tight SYCP3 association and are broad in width.

### SYCP1 mainly occupies highly-opened meiotic chromatin regions with abundant SYCP3 and cohesin association

**Figure 5.**
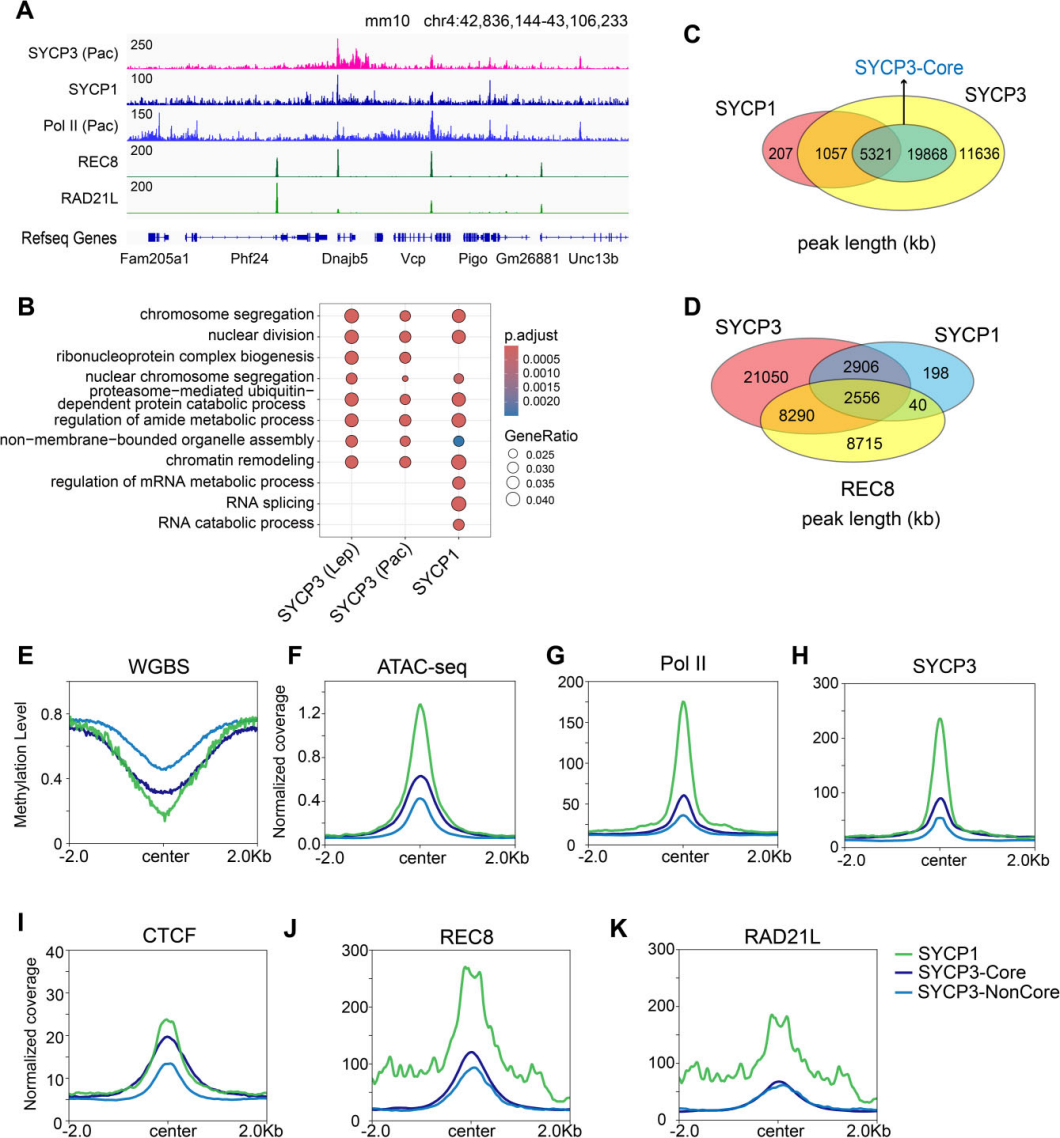
Identification of SYCP1 binding sites in pachytene spermatocytes. (**A**) IGV genome browser snapshots of occupancy of SYCP3, SYCP1, Pol II, REC8, and RAD21L in mouse spermatocytes. (**B**) Gene Ontology analysis on genes with promoters containing SYCP3 peaks in leptotene, SYCP3 peaks in pachytene, and SYCP1 peaks in pachytene, respectively. (**C**) Venn diagrams showing the overlap of SYCP1, SYCP3, and SYCP3-Core in spermatocytes. (**D**) Venn diagrams showing the overlap of SYCP3, SYCP1, and REC8 in spermatocytes. (**E**, **F**) Intensity of DNA methylation and chromatin accessibility of SYCP3-Core, SYCP3-NonCore, and SYCP1 peaks in spermatocytes by WGBS and ATAC-seq. (**G**–**K**) Intensity of chromatin association of Pol II (**G**), SYCP3 (**H**), CTCF (**I**), REC8 (**J**), and RAD21L (**K**) at SYCP3-Core, SYCP3-NonCore, and SYCP1 peaks (2 kb flanking center) in spermatocytes.

To further explore meiotic chromatin structure by SC assembly, we examined the chromatin occupancy of SYCP1, which bridges LE and CE structures in pachytene spermatocytes (Fig. 5A). Compared to SYCP3, SYCP1 has a different chromatin distribution pattern (Supplementary Fig. S3B) and a different type of associated genes (Fig. 5B). Notably, the majority of SYCP1-occupied genomic regions were identified in SYCP3-binding sites, especially SYCP3-Core sites (Fig. 5C). REC8, the subunit of the meiotic cohesin complex, is a major component of meiosis-specific chromosome axes, and our analysis showed that REC8 has similar genome distribution to SYCP3 (Fig. 5A). Additionally, both SYCP3 and SYCP1 were enriched at REC8 peaks (Supplementary Fig. S5A), and a significant amount of SYCP1 peaks localized in REC8-occupied regions (Fig. 5D). Profiling of SYCP1 along genic regions revealed its abundance at promoter and enrichment at gene body (Supplementary Fig. S5B), suggesting its attachment to open chromatin regions. Therefore, we compared chromatin properties at SYCP3-Core sites, SYCP3-NonCore sites, and SYCP1 peaks. Notably, we observed the lowest DNA methylation (Fig. 5E), the highest chromatin accessibility (Fig. 5F), the strongest Pol II, SYCP3, and CTCF binding (Fig. 5G–I) at SYCP1 peaks compared to SYCP3-Core and SYCP3-NonCore sites. Meiosis-specific cohesin complex plays a critical role in the assembly of normal axial elements and meiotic recombination [47]. Interestingly, we noticed high abundance of the REC8 and RAD21L subunits of the meiotic cohesin complex [40] at SYCP1 peaks (Fig. 5J and K), suggesting cooperation of SYCP1 and cohesin in bridging LE and CE structures. The HOMER algorithm of SYCP1 peaks identified enriched motifs of several transcription factors including NF-Y, NRF1, and KLF10 (Supplementary Fig. S5C).

Taken together, we show that the majority of SYCP1-binding sites at meiotic chromatin is a subset of SYCP3 peaks with lower DNA methylation, higher chromatin accessibility, and enriched meiotic cohesin complex, implying unique structural functions of these meiotic chromatin regions in spermatocytes.

### Transposable elements and pachytene piRNA (PIWI-interacting RNA) loci are enriched in SYCP1/3-occupied regions in spermatocytes

**Figure 6.**
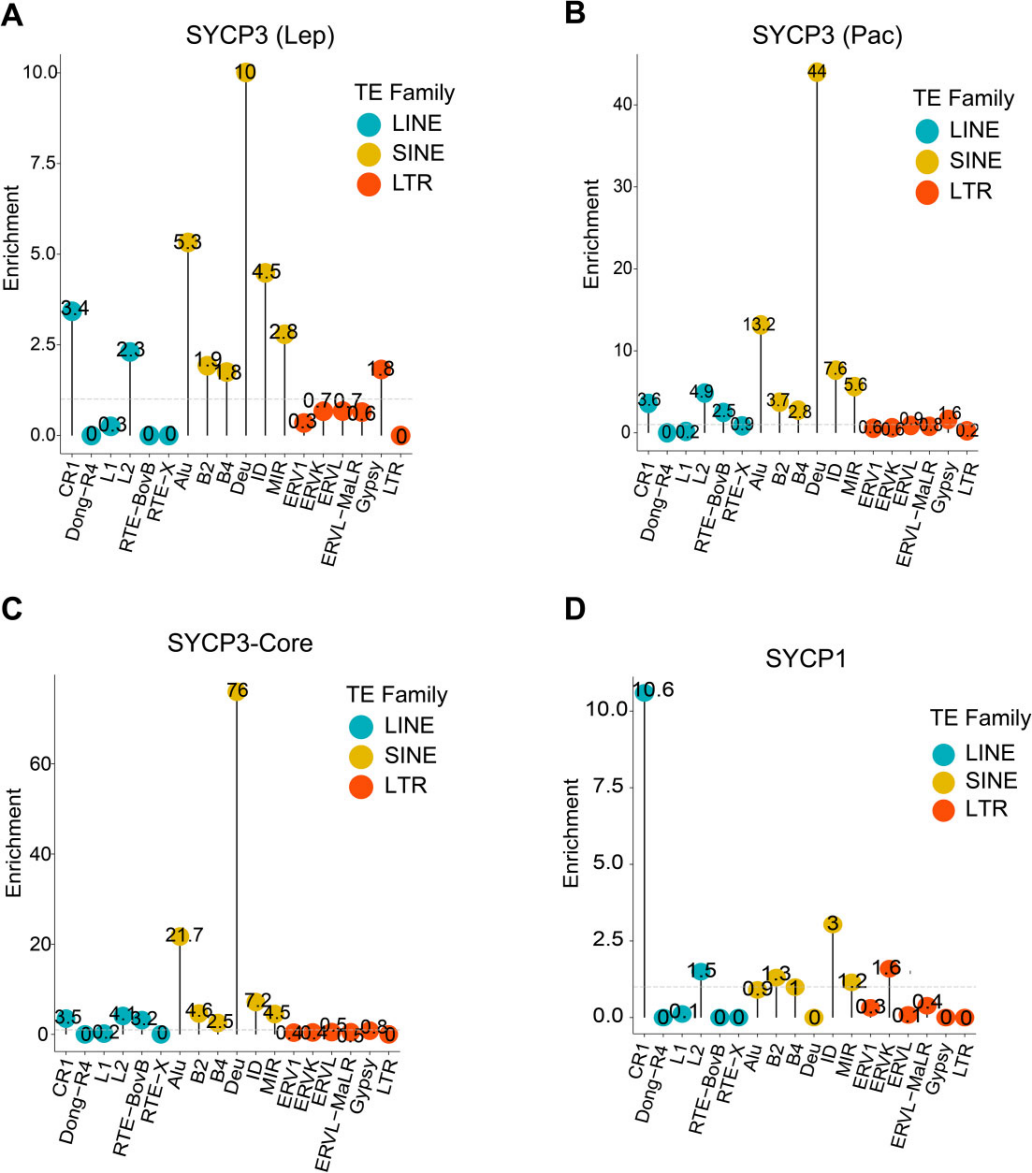
Characterization of SYCP3/1 binding at repetitive elements in spermatocytes. Diagram showing the enrichment of TE families, including SINEs, LINEs, and LTR in SYCP3-Lep (**A**), SYCP3-Pac (**B**), SYCP3-Core (**C**), and SYCP1 peaks (**D**). Dashed lines indicate an enrichment value of 1 representing no enrichment.

TEs make up about half of mammalian genomes, shape genome architecture through association with various chromatin regulators, and play important regulatory roles in the male germline [48]. It has been reported that these repetitive elements are tethered to meiotic chromosome axis [49–51]. Our analyses showed that different classes of TE were enriched in SYCP3 and SYCP1 binding sites in pachytene spermatocytes, relative to fractions of TE classes in mouse genome (Supplementary Fig. S6A). Further enrichment analysis on TE families showed significant enrichment of Deu and Alu elements of short interspersed nuclear elements (SINEs) at SYCP3 peaks during meiosis especially at SYCP3-Core sites (Fig. 6A–C, *P* values were summarized in Supplementary Table S4). Distinct from SYCP3, SYCP1 is mainly associated with CR1 elements of long interspersed nuclear elements (LINEs) (Fig. 6D). To identify chromatin signatures of Deu, Alu, and CR1 elements during meiosis, we profiled epigenetic modifications of TE subfamilies in spermatocytes. The three elements (Deu, Alu, and CR1) are characterized by high CTCF occupancy, high histone acetylation, and relatively repressed chromatin regions, respectively (Supplementary Fig. S6B). Alu elements are simple sequence repeats accumulated mainly at gene-rich regions [52] and were recently implicated in mediating long-range chromatin interactions by forming RNA duplexes with complementary sequences [53]. Therefore, RNA–RNA interaction mechanisms may exist at the repetitive elements to recruit meiotic axial proteins and mediate meiotic chromosome axis formation as well as homologous chromosome recognition. Annotation of SYCP3-enriched and SYCP1-enriched repetitive elements showed that these regions are mainly localized at promoter, intron, and distal regions (Supplementary Fig. S6C). Moreover, these regions have globally lower Pol II association than peaks at non-TE regions (Supplementary Fig. S7A–C). Previously, we identified specific occupancy of BTBD18 at a subset of pachytene piRNA loci, classified piRNA precursors into three populations: PPBD (pachytene piRNA precursor-BTBD18 dependent), PPBI (pachytene piRNA precursor-BTBD18 independent), and PrePP (pre-pachytene piRNA precursor), and observed that nuclear localization of BTBD18 was in proximity with SYCP3 stretches [28]. Here, our analysis showed that SYCP3 and SYCP1 proteins are more enriched at genomic regions of pachytene piRNA precursors including PPBD and PPBI loci than PrePP loci (Supplementary Fig. S8A and B). Taken together, our results indicate that TEs and pachytene piRNA loci are associated with SC to form unique meiotic chromatin structure.

### SYCP3 exhibits positive correlation with histone H3.3 at sex chromosomes in spermatocytes

**Figure 7.**
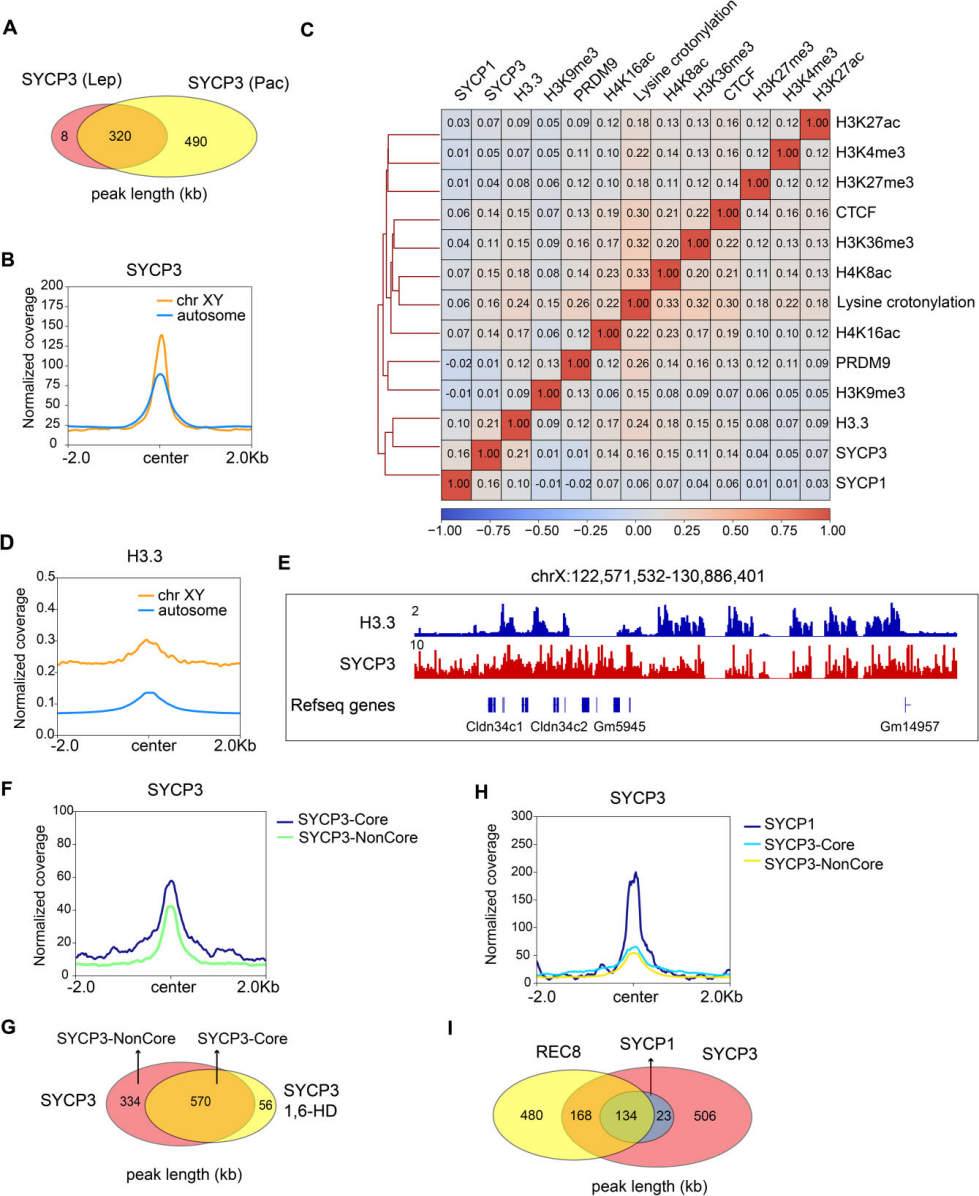
Characterization of SYCP3/1 distribution at sex chromosomes in spermatocytes. (**A**) Venn diagrams showing the overlap of SYCP3 peaks at sex chromosomes in pachytene and leptotene spermatocytes. (**B**) Intensity of SYCP3 occupancy at sex chromosomes and autosomes in pachytene spermatocytes (2 kb flanking peak center). (**C**) Heatmap of pairwise Spearman correlation of chromatin association of SYCP3/1, H3.3, histone marks, and CTCF at sex chromosomes of spermatocytes. (**D**) Intensity of H3.3 occupancy at SYCP3-occupied sites from sex chromosomes and autosomes in pachytene spermatocytes (2 kb flanking peak center). (**E**) IGV genome browser snapshot of chromatin occupancy of SYCP3 and H3.3 at X chromosome in mouse pachytene spermatocytes. (**F**) Intensity of SYCP3 occupancy at SYCP3-Core and SYCP3-NonCore sites at sex chromosomes in pachytene spermatocytes (2 kb flanking peak center). (**G**) Venn diagram showing the overlap of SYCP3 peaks at sex chromosomes without (SYCP3) and with 1,6-HD treatment (SYCP3-1,6-HD) in pachytene spermatocytes. (**H**) Intensity of SYCP3 occupancy at SYCP1, SYCP3-Core, and SYCP3-NonCore sites at sex chromosomes in pachytene spermatocytes (2 kb flanking peak center). (**I**) Venn diagrams showing the overlap of SYCP3 , SYCP1, and REC8 peaks at sex chromosomes in pachytene spermatocytes.

In our above analysis, we observed no obvious colocalization of SYCP3 and Pol II at sex chromosomes, along with weak immunofluorescence signal for Pol II at sex chromosomes (Fig. 2E). To further investigate the characteristics of SYCP3 distribution at sex chromosomes, we examined SYCP3 signals enriched at sex chromosomes and found that these peaks constituted 1.32% (121/9015) of total peaks at leptotene stage and 2.32% (885/37226) at pachytene stage. Notably, the peaks at sex chromosomes in the two stages were highly overlapped (Fig. 7A). We also noticed that the intensity of SYCP3 at sex chromosomes was higher than that at autosomes (Fig. 7B). Subsequently, we classified SYCP3 peaks enriched in sex chromosomes at pachytene stage into three groups according to the intensity of SYCP3 (Supplementary Fig. S9A). Generally, stronger SYCP3 occupancy was correlated with higher chromatin accessibility by ATAC-seq and lower DNA methylation by WGBS (Supplementary Fig. S9B and C). To further explore epigenetic regulation of SYCP3 occupancy at sex chromosomes, we performed correlation analysis of SYCP3 and other modifications and found relatively higher correlation between H3.3 and SYCP3 (Fig. 7C). Moreover, H3.3 enrichment was higher at SYCP3 peaks at sex chromosomes than autosomes (Fig. 7D), and H3.3 colocalized with SYCP3 peaks at sex chromosomes by manual visualization (Fig. 7E). These results suggest that H3.3 may play an important role in orchestrating SYCP3 occupancy at sex chromosomes.

Furthermore, we analyzed the enrichment of SYCP3 at SYCP3-Core and SYCP3-NonCore sites within sex chromosomes and observed stronger SYCP3 binding at the SYCP3-Core sites (Fig. 7F and G). SYCP1-binding sites at sex chromosomes were also examined. The result showed that the enrichment of SYCP3, CTCF, RAD21L, and REC8 were all stronger at SYCP1-binding sites than SYCP3-Core/Noncore sites (Fig. 7H and I, and Supplementary Fig. S9D–F). Additionally, DNA methylation level was the lowest in SYCP1-binding sites (Supplementary Fig. S9G), while similar signal intensities of H3.3 were identified at SYCP1-binding sites and SYCP3-Core/Noncore sites (Supplementary Fig. S9H).

In summary, our study documents the binding of SYCP3 at active chromatin regions in mouse spermatocytes and demonstrate its dynamics and regulation by transcription and other chromatin events during meiosis. Our study sheds light on molecular mechanisms underlying meiotic chromosome axis establishment and regulation. Further exploration using knockout mouse models of meiotic axial proteins and epigenetic modifiers will provide valuable information on meiotic chromatin architecture and crosstalk among meiotic chromatin events.

## Discussion

SYCP3 is widely known as the critical component of the axial element of SC in meiotic cells. SYCP3 deficiency in male mice leads to sterility and failed synapsis [4]. SYCP1 protein was used to label SC, and short stretches of SYCP1 fibers and axial interruptions were observed upon *Sycp3* depletion, indicating that SYCP3 partially participates in establishing meiotic chromosome axis structure. Previous super-resolution imaging identified several epigenetic marks emerging laterally from the SC and indicated high chromatin accessibility away from the SC [10]. However, little is known about genome-wide SYCP3 distribution in meiosis and its regulation.

**Figure 8.**
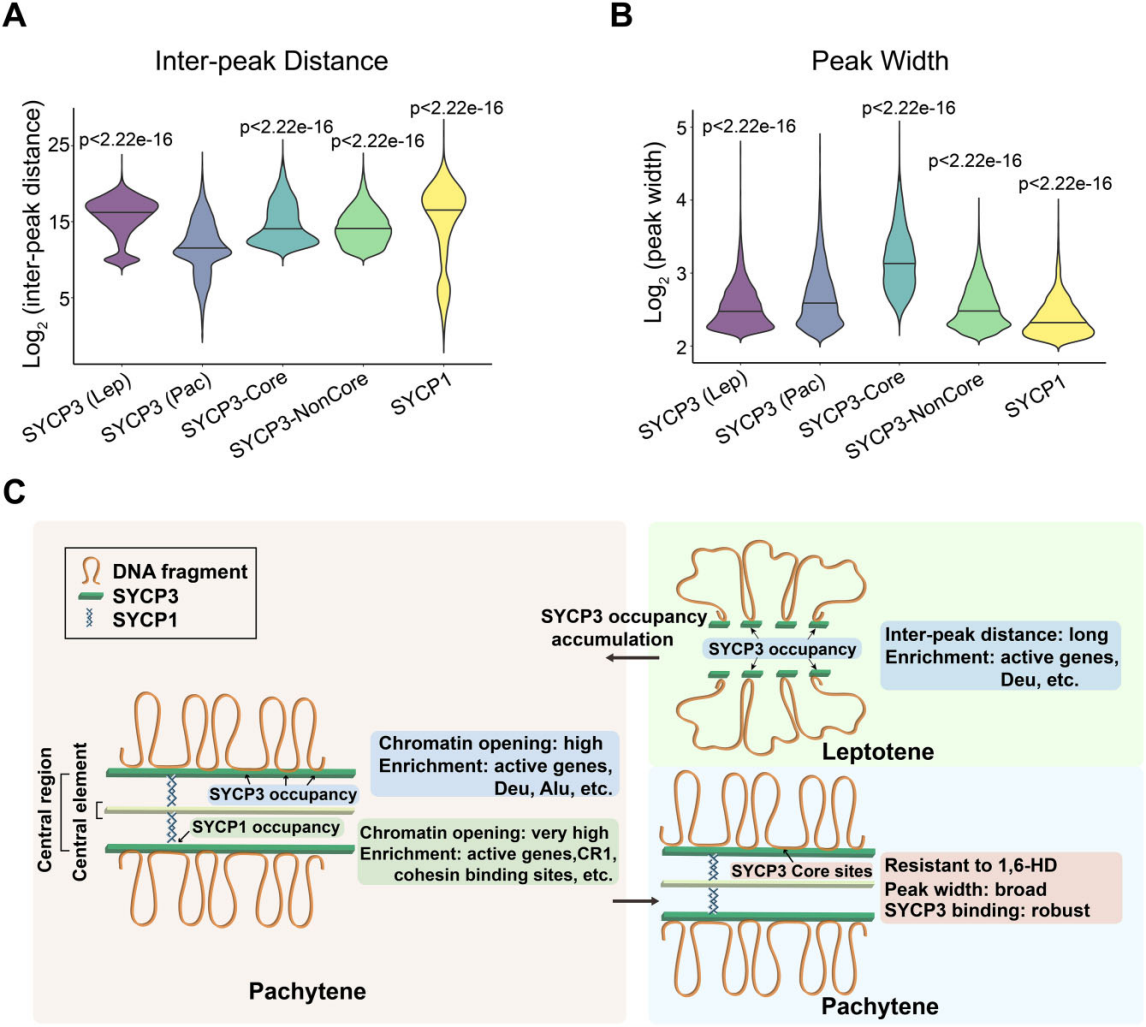
Schematic of how SYCP3 is involved in the organization of meiotic chromatin architecture. Violin plots comparing log_2_(inter-peak distance) (**A**) and log_2_(peak width) (**B**) of indicated peaks. SYCP3 (Pac) group was used as a reference to calculate *P* values by the Wilcoxon rank-sum test. (**C**) Model illustrating meiotic chromatin architecture in leptotene and pachytene spermatocytes, with occupancy of SYCP3/1 and related chromatin features indicated.

In this study, we identified chromatin occupancy of SYCP3 in leptotene and pachytene spermatocytes, SYCP3-Core sites retained upon treatment of 1,6-HD, which appears to disrupt larger SYCP3 fibrous assemblies, and SYCP1 occupancy at meiotic chromatin in pachytene spermatocytes. When we determined inter-peak distances (Fig. 8A) and width of peaks (Fig. 8B), we observed that: (a) SYCP3 occupancy in the leptotene stage had greater inter-peak distances and narrower peak width than SYCP3 occupancy in the pachytene stage, demonstrating gradual accumulated SYCP3 at chromatin during meiotic progression; (b) SYCP3-Core sites have broader peak width than SYCP3-NonCore sites, in agreement with stability of SYCP3 occupancy at SYCP3-Core sites; (c) SYCP1 occupancy had greater inter-peak distances and narrower peak width than SYCP3 occupancy in pachytene stage, supporting that SYCP1 peaks are a subset of SYCP3 peaks and SYCP1’s major function in connecting LE and CE respectively. Our exploration of chromatin signatures of these peaks provides useful information on the dynamic organization of meiotic chromosomes by meiotic axial proteins (Fig. 8C). Generally, at pachytene-stage meiosis, SYCP3 mainly occupies chromatin regions including transcriptionally active sites, Deu and Alu elements, while SYCP1 mainly co-localizes with SYCP3 at a subset of regions with open chromatin and high cohesin enrichment to connect meiotic chromosome axes with CE. In this way, a local active chromatin environment is created at meiotic chromosome axes, facilitating homologous chromosome recombination. In contrast to the pachytene stage, SYCP3 in the leptotene stage initially loosely associates with chromatin. Despite the significant accumulation of SYCP3 during meiotic progression, not all SYCP3 occupancy can be maintained during 1,6-HD treatment. Those SYCP3-stably-occupied regions, SYCP3-Core sites, had stronger SYCP3 binding that was broader. Please see Supplementary Fig. S10 for distribution maps of mouse chromosomes for SYCP3, SYCP1, γH2A.X, Pol II, and DMC1 peaks in mouse spermatocytes.

It is interesting to determine how SYCP3 occupancy and epigenetic modifications interact with each other in meiotic chromatin. SYCP3 has an intrinsic activity to directly associate with naked DNA, and we report that SYCP3 also associates with Pol II and occupies transcriptionally active regions. The association of SYCP3 at SINE repeats suggests the involvement of RNA polymerase III in meiotic chromatin organization because transcription of SINE repeats is driven by RNA polymerase III. Mechanistically, SYCP3 may help condense meiotic chromosomes by forming supramolecular protein assemblies with epigenetic and transcriptional modulators to create a local active chromatin environment. However, the detailed structures of meiotic chromosome axes and how SYCP3 cooperates with other meiotic axial proteins like SYCP2 to organize meiotic chromatin remain to be determined. In addition, posttranslational modifications of meiotic axial proteins [54], such as SYCP3 phosphorylation, should also play an important role in this process. Human SYCP3 may also colocalize with Pol II and share similar genome-wide occupancy pattern to mouse SYCP3. Notably, components and activities of repetitive elements in primates are different from rodents, so TEs enriched in SYCP1/3 occupied regions and related molecular mechanisms may be different in humans. However, it remains unclear how repetitive elements are involved in meiotic chromatin organization. Enrichment of SYCP1/3 at repetitive elements hints that these sequences may facilitate homology recognition during meiosis and genetic recombination. Some of the repetitive elements may function as enhancers in cells [53, 55], and through anchoring to chromosome axis, these elements may participate in transcriptional regulation by forming physical proximity with distant gene expression cassettes, which are also anchored to chromosome axis. However, it was also proposed that due to divergent sequences of repetitive elements, these repetitive sequences may antagonize recombination through impeding strand exchanges to ensure chromosome stability [56]. We also observed association of pachytene piRNA clusters with SYCP3/SYCP1, and this interaction may facilitate recombination events at these piRNA loci to diversify piRNA repertoire for fast evolving. What’s more, it will be interesting to identify SYCP3 occupancy during meiosis in females, because there are differences between meiotic chromatin architecture in oocyte and spermatocyte, and there are no XY body in the oocyte. However, our test was not successful in examining SYCP3 occupancy with as few as <1000 spermatocytes using our CUT&Tag protocol, which was proven successful to profile Pol II and H3K4me3 using 100 oocytes [57], so improvement of efficiency of tagmentation or development of more efficient antibodies against SYCP3 will be helpful for further studies. Additionally, SYCP3 may also function in cancer cells and modulate genome integrity [58].

## Supplementary Material

gkaf460_Supplemental_Files

## Data Availability

CUT&Tag data generated in this study has been deposited to the GEO database under the accession number GSE237315 (https://ncbi.nlm.nih.gov/geo/query/acc.cgi?acc=GSE237315). Public mouse ChIP-seq data were obtained from GSE61613 (H3K9me3 and PRDM9), GSE49624 (H3K4me3 and H3K27ac), GSE49621 (H3K27me3), GSE32663 (K-Cro), GSE31039 (H3K36me3), GSE132054 (CTCF, REC8, and RAD21L), GSE69946 (H4K8ac and H4K16ac). DMC1–SSDS data were obtained from GSE262343 (leptotene/zygotene) and GSE35498 (pachytene). Public ATAC-seq data of mouse spermatocytes were obtained from GSE81470. Public WGBS data of mouse spermatocytes were obtained from GSE100220.
