# Supplementary material for: Deciphering meiotic chromatin organization by SYCP3
Source: Nucleic Acids Res. 2025 Jun 9;53(11):gkaf460. doi: 10.1093/nar/gkaf460 (PMC12146848; doi:10.1093/nar/gkaf460)
Supplement: gkaf460_Supplemental_Files [file gkaf460_supplemental_files.zip › Revised_Supplementary_Figures_clean.docx]

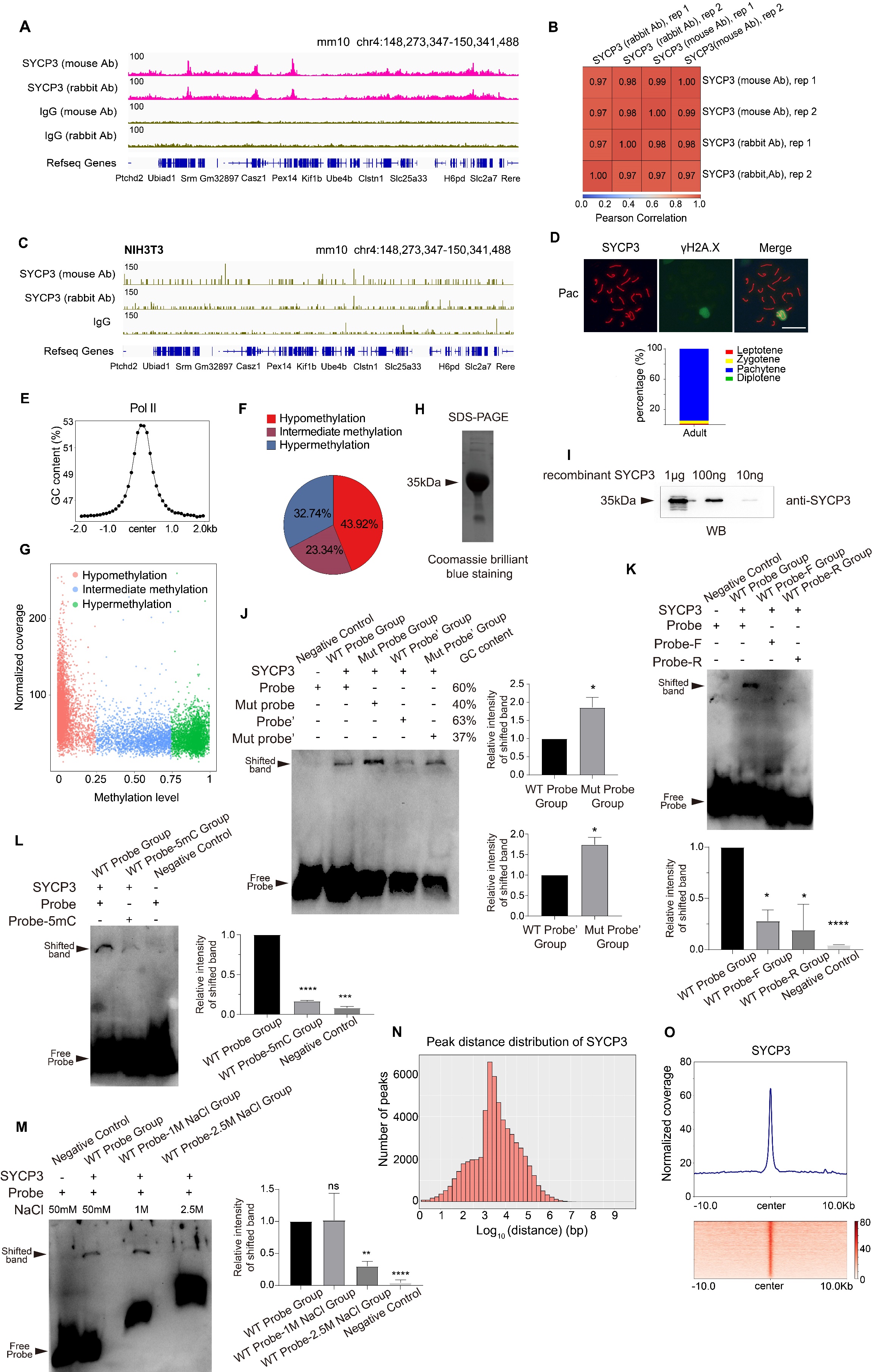


**Supplementary Figure 1. Distribution of SYCP3 occupancy in meiotic chromatin.**

(A) IGV genome browser snapshots of SYCP3 occupancy in spermatocytes from 4-week-old male mice with IgG as negative control. Anti-SYCP3 antibodies of mouse and rabbit origin were used in CUT&Tag assay. (B) Pearson correlation analysis of SYCP3 occupancy using anti-SYCP3 antibodies of mouse (mouse Ab) and rabbit (rabbit Ab) origin in spermatocytes from 4-wk-old mice. (C) Visualization of SYCP3 enrichment at chromatin in NIH3T3 cells via IGV genome viewer, with IgG used as negative control. (D) Representative chromosome spread of pachytene spermatocyte with anti-SYCP3 and anti-γH2A.X antibody staining, and bar graph of meiotic stages of purified spermatocytes from adult testicular cells. Scale bar, 20 μm. (E) Density plot of GC content around the peak center of Pol II (± 2 kb) in pachytene spermatocytes. Each dot represents mean GC content using a 100 bp-wide rolling window. (F) Pieplot showing DNA methylation status of SYCP3 peaks in pachytene spermatocytes. (G) Diagram showing SYCP3 binding activity in three annotated DNA methylation regions including hypo, intermediate, and hypermethylation. Each dot represents individual SYCP3 peaks in pachytene spermatocytes. (H) Coomassie Brilliant Blue staining of recombinant SYCP3 protein. (I) Immunoblotting of recombinant SYCP3 protein. (J) EMSA assay showing the binding activity of the double-stranded labeled DNA and recombinant SYCP3 protein. Probe and Probe’ are mouse sequences derived from SYCP3 CUT&Tag peak summit in pachytene spermatocyte. Mut sequence was obtained through G/C-to-A/T and A/T-to-G/C substitution of WT sequence. WT Probe, forward: 5’-ACAGTCCTGGTGATTGAACTCCGGCCCTGGGCATGCCAGGCGAGCACTCCACTCTTTGAG-3’; reverse: 5’- CTCAAAGAGTGGAGTGCTCGCCTGGCATGCCCAGGGCCGGAGTTCAATCACCAGGACTGT-3’. Mut Probe, forward: 5’- GTGACTTCAACAGCCAGGTCTTAATTTCAAATGCATTGAATAGATGTCTTGTCTCCCAGA-3’; reverse: 5’- TCTGGGAGACAAGACATCTATTCAATGCATTTGAAATTAAGACCTGGCTGTTGAAGTCAC-3’. WT Probe’, forward: 5’- TAGGTTGGCTAGTAGGGGTGGGGTCTGGGCTGTGGTGGCAGAGTCCTCTTGGGAGGGCTC-3’; reverse: 5’- GAGCCCTCCCAAGAGGACTCTGCCACCACAGCCCAGACCCCACCCCTACTAGCCAACCTA-3’. Mut Probe’, forward: 5’-CGAACCAATCGACGAAAACAAAACTCAAATCACAACAATGAGACTTCTCCAAAGAAATCT-3’; reverse: 5’- AGATTTCTTTGGAGAAGTCTCATTGTTGTGATTTGAGTTTTGTTTTCGTCGATTGGTTCG-3’. Bar graph (right) showing the ratio of the gray value of samples from different lanes to that of the WT Probe Group. n = 2 per group. (K-L) EMSA showing the binding activity of the single-stranded labeled DNA (K) and 5-methylated double-stranded labeled DNA (L) with WT Probe sequence and recombinant SYCP3 protein. Bar graphs showing the ratio to the gray value of the WT Probe Group. Probe-F: 5’-ACAGTCCTGGTGATTGAACTCCGGCCCTGGGCATGCCAGGCGAGCACTCCACTCTTTGAG-3’. Probe-R: 5’- CTCAAAGAGTGGAGTGCTCGCCTGGCATGCCCAGGGCCGGAGTTCAATCACCAGGACTGT-3’. Probe-5mC, forward: 5’-A/^5m^C/AGT/^5m^C/^5m^C/TGGTGATTGAA/^5m^C/T/^5m^C/^5m^C/GG/^5m^C/^5m^C/^5m^C/TGGG/^5m^C/ATG/^5m^C/^5m^C/AGG/^5m^C/GAG/^5m^C/A/^5m^C/T/^5m^C//^5m^C/A/^5m^C/T/^5m^C/TTTGAG-3’; reverse: 5’-^5m^C/T/^5m^C/AAAGAGTGGAGTG/5mC/T/^5m^C/G/^5m^C/^5m^C/TGG/^5m^C/ATG/^5m^C/^5m^C/^5m^C/AGGG/^5m^C/^5m^C/GGAGTT/^5m^C/AAT/^5m^C/A/^5m^C/^5m^C/AGGA/^5m^C/TGT-3’. n = 2 per group. (M) EMSA result showing the binding activity of the WT Probe and recombinant SYCP3 protein at different concentration of NaCl. Bar graph showing the relative gray value of different groups (right). The concentration of NaCl added to the system and the location of the shifted bands have been showed. n = 2 per group. *p* value was calculated by the Student’s t-test. (N) Distribution of peak distances between nearest SYCP3 peaks in pachytene spermatocytes. (O) Density plot of SYCP3 peak distribution (center ± 10.0 kb) in pachytene spermatocytes.


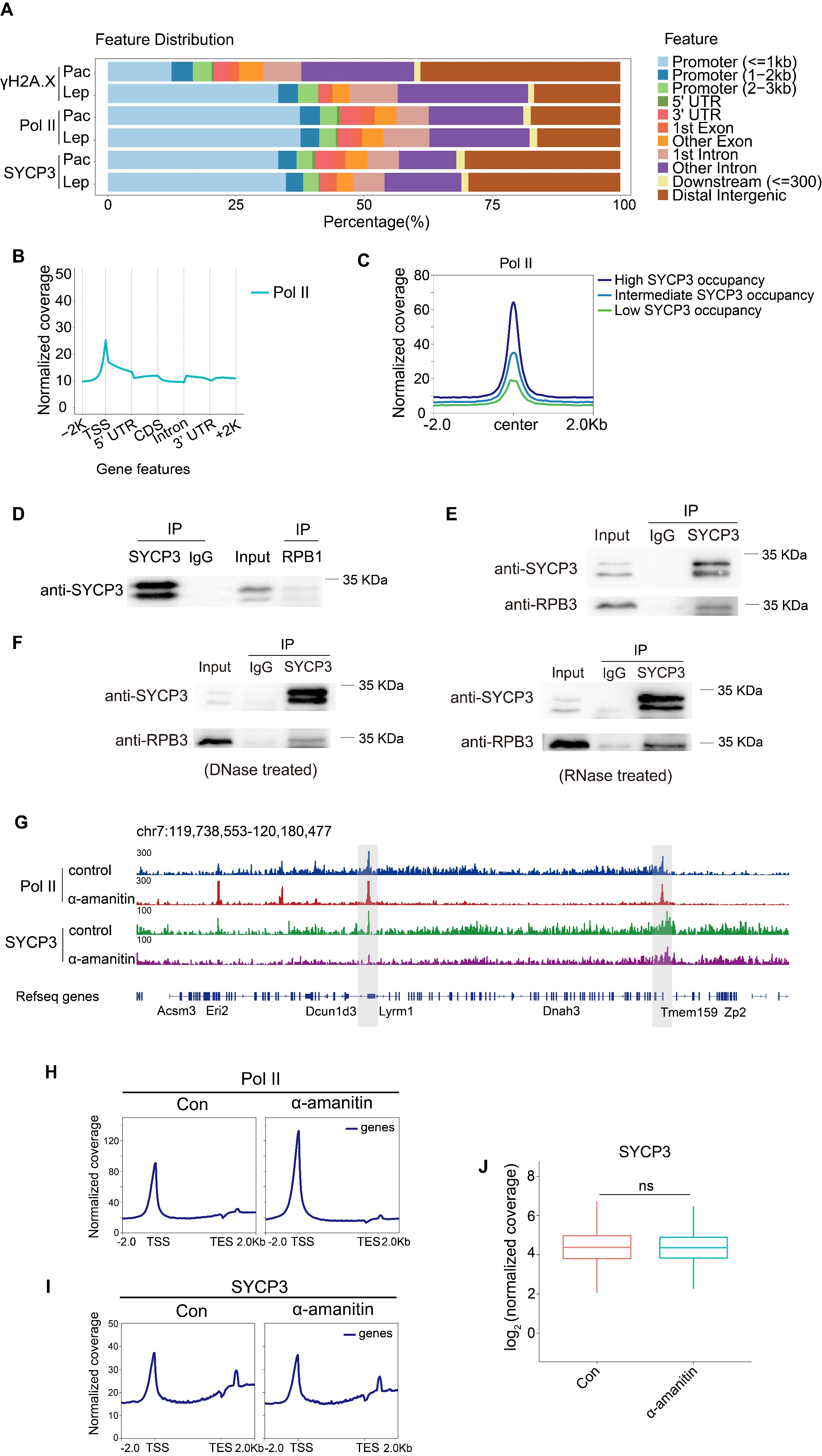


**Supplementary Figure 2. Relationship of SYCP3 and Pol II in spermatocytes.**

(A) Feature distribution map of the binding of SYCP3, Pol II and γH2A.X at meiotic chromosome in mouse leptotene/pachytene spermatocytes. (B) Diagram showing Pol II distribution along genic regions in pachytene spermatocytes. (C) Read density plot showing distribution of Pol II at genomic regions with high/intermediate/low SYCP3 occupancy (peak center ± 2.0 kb). (D) Immunoblotting of SYCP3 after immunoprecipitation with IgG, anti-SYCP3 antibody and anti-RPB1 antibody from adult testicular lysate, using Input without immunoprecipitation as positive control. RPB1 is the largest subunit of Pol II. (E) Immunoblotting of SYCP3 and RPB3 after immunoprecipitation with IgG or anti-SYCP3 antibody from adult testicular lysate, using Input without immunoprecipitation as positive control. (F) Immunoblotting of SYCP3 and RPB3 after immunoprecipitation with IgG or anti-SYCP3 antibody. 5 μg/L DNase I or RNase A was used to treat testicular lysate for 30min at room temperature, followed by overnight incubation of the reaction with indicated antibodies. (G) Genome browser view of Pol II and SYCP3 enrichment before and after α-amanitin treatment via IGV genome viewer. (H-I) Intensity of Pol II (H) and SYCP3 (I) signals at SYCP3-associated genes (2 kb flanking TSS and TES) in the absence (left) or presence (right) of α-amanitin in cultured spermatocytes. (J) Boxplot indicating no change of SYCP3 intensity at gene promoters upon α-amanitin treatment. *p* value was calculated by the Wilcoxon rank-sum test.


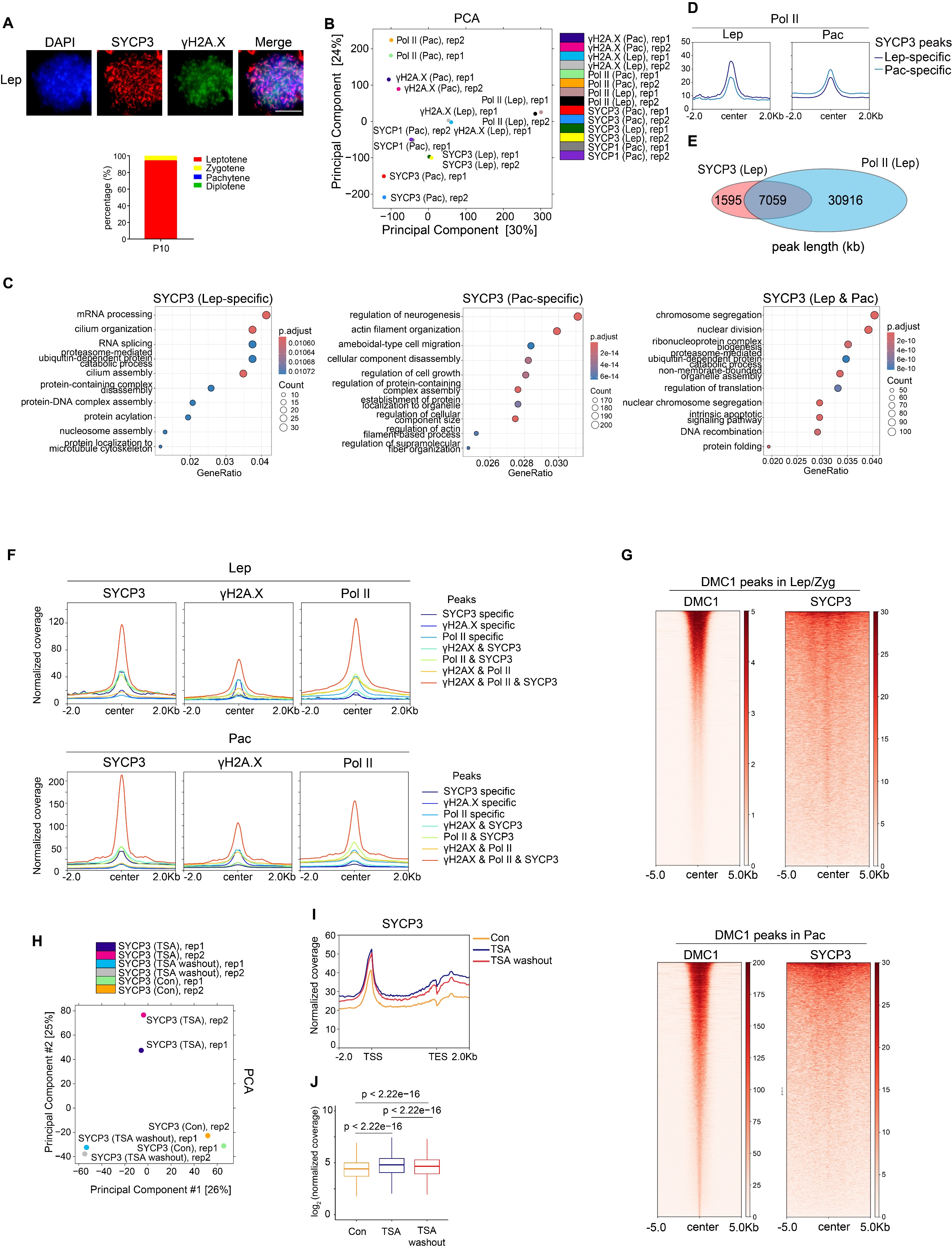


**Supplementary Figure 3. Chromatin binding of SYCP3, Pol II and γH2A.X at leptotene and pachytene stages.**

(A) Representative image of leptotene spermatocyte in chromosome spread analysis with SYCP3 and γH2A.X staining, and bar graph demonstrating meiotic stages of purified spermatocytes from P10 testicular cells. Scale bar, 20 μm. (B) Principal Component Analysis (PCA) of chromatin occupancy of SYCP3/1, Pol II and γH2A.X in leptotene and pachytene spermatocytes. (C) Gene ontology analysis of genes with promoters containing stage-specific or overlapping SYCP3 peaks in leptotene and pachytene spermatocytes. (D) Density plot of Pol II enrichment at stage-specific SYCP3 peaks in leptotene and pachytene spermatocytes. (E) Venn diagram showing the overlap of SYCP3 and Pol II peaks in leptotene spermatocytes. (F) Intensity of the signals of SYCP3, γH2A.X and Pol II at SYCP3/γH2A.X/Pol II-specific and overlapped peaks (2 kb flanking peak center) in leptotene and pachytene spermatocytes. Pol II & SYCP3 represents the overlapping peaks of Pol II and SYCP3. γH2A.X & Pol II represents the overlapping peaks of γH2A.X and Pol II. γH2A.X & Pol II & SYCP3 represents the overlapping peaks of γH2A.X, Pol II and SYCP3. (G) SYCP3 occupancy at DMC1-marked DSB hotspots in leptotene/zygotene (GSE262343) and pachytene (GSE35498) stages by DMC1-SSDS. (H) PCA of chromatin occupancy of SYCP3 in control, TSA and TSA washout groups of pachytene spermatocytes. (I) Intensity of SYCP3 occupancy at SYCP3-binding genic regions (2 kb flanking TSS and TES) in Con, TSA and TSA washout groups of pachytene spermatocytes. (J) Boxplot shows quantification of SYCP3 occupancy at SYCP3-binding genic regions (2 kb flanking TSS and TES) in Con, TSA and TSA washout groups of pachytene spermatocytes.


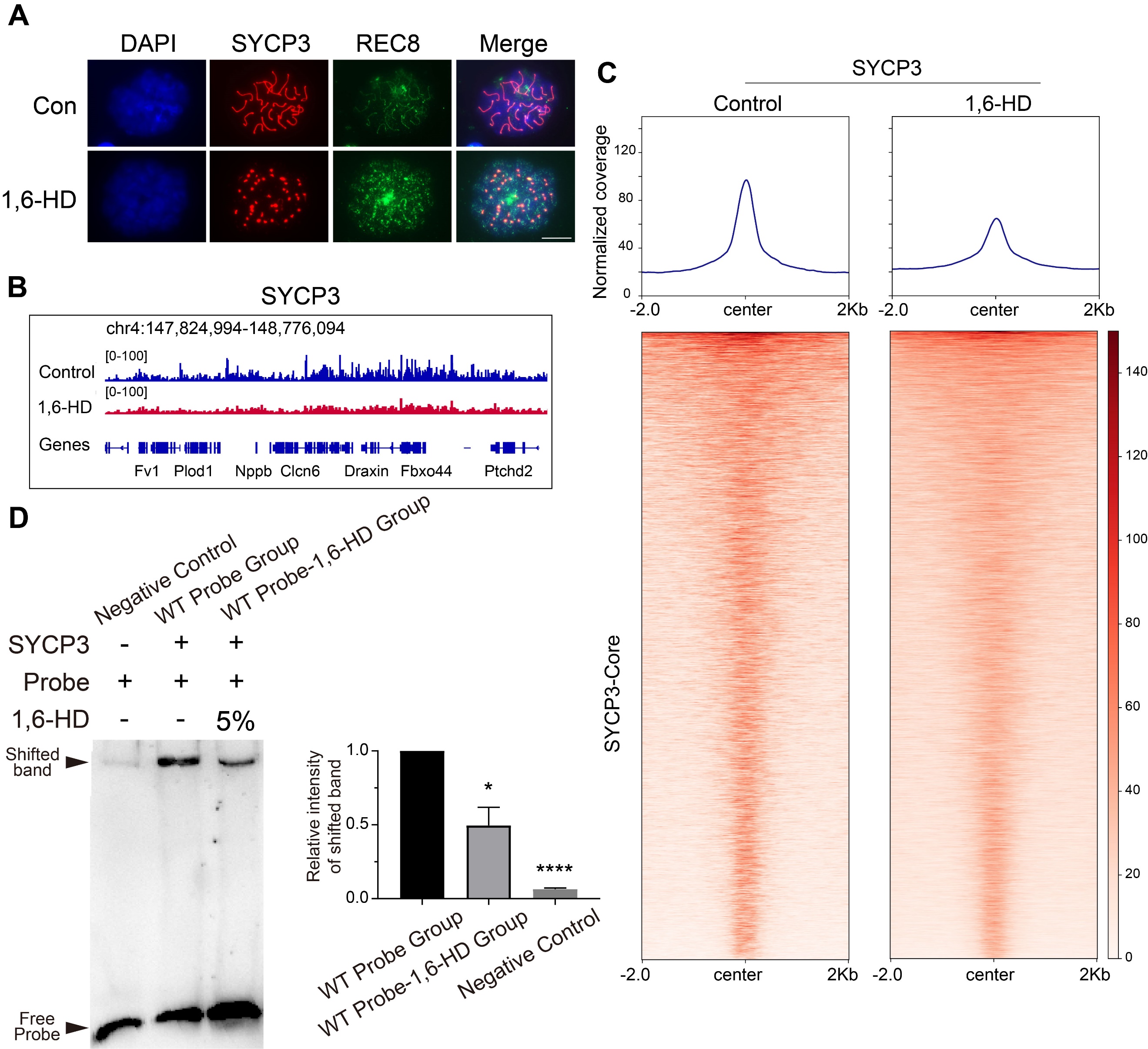


**Supplementary Figure 4. Characteristics of SYCP3-Core sites in spermatocytes on HFD.**

(A) Immunostaining of SYCP3 (red) and REC8 (green) using chromosome spread of pachytene spermatocytes with or without 1,6-HD treatment. DNA was stained with DAPI (blue). Scale bar, 10 μm. (B) Genome browser view of SYCP3 enrichment without and with 1,6-HD treatment via IGV genome viewer. (C) Density plot of SYCP3 occupancy at SYCP3-Core sites (center ± 2.0 kb) in pachytene spermatocytes with or without 1,6-HD treatment. (D) EMSA assay showing the binding activity of the double-stranded labeled WT Probe and recombinant SYCP3 protein with/without 1,6-HD. Bar graph (right) showing the ratio of the gray value of samples from different lanes to that of the WT Probe Group. The concentration of 1,6-HD added to the system and the location of the shifted bands have been showed. n = 2 per group. *p* value was calculated by the Student’s t-test.


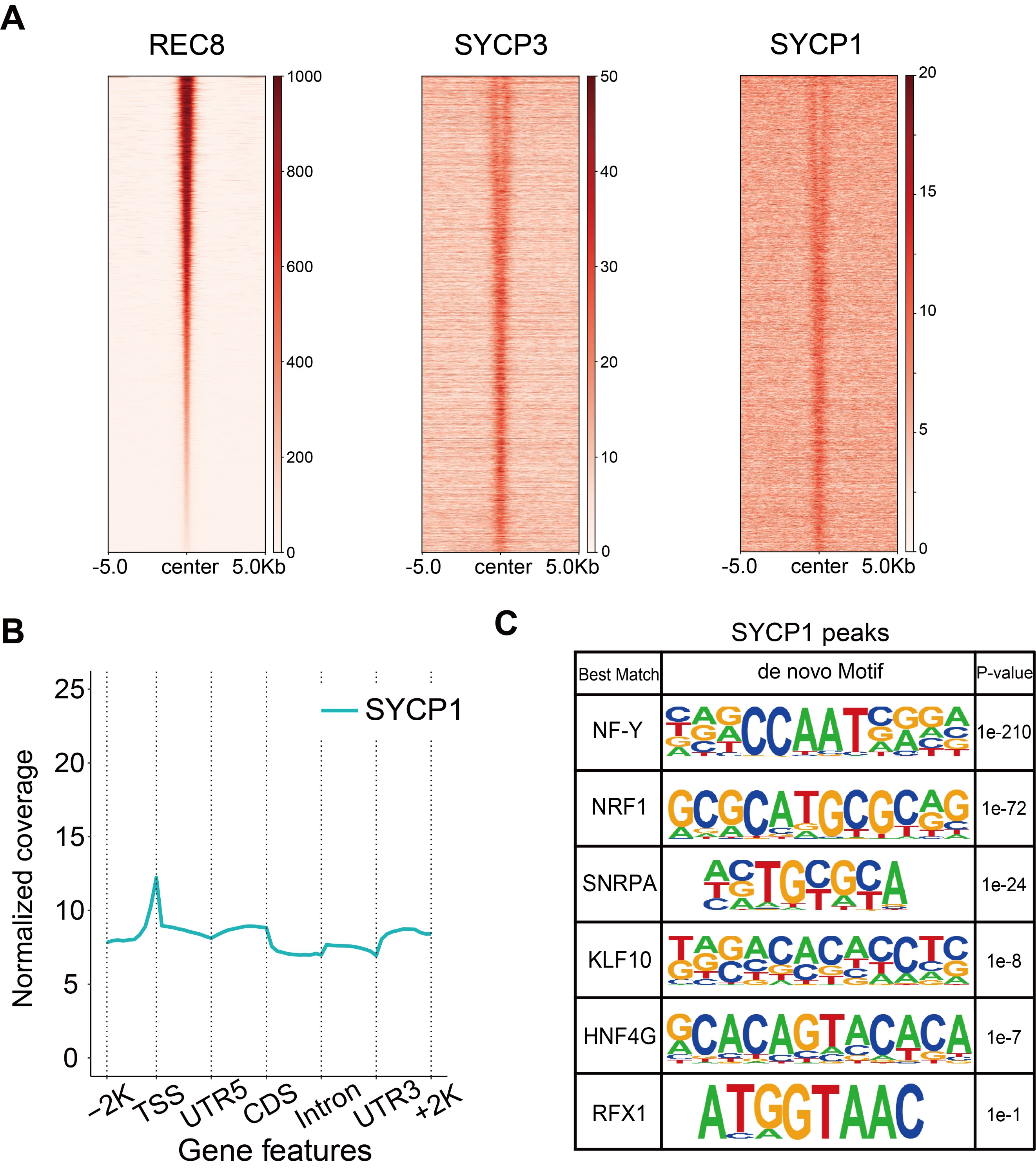


**Supplementary Figure 5. Association of SYCP1 at meiotic chromatin.**

(A) Density plot of peak distribution (center ± 5.0 kb) of SYCP3 and SYCP1 at REC8 peaks in pachytene spermatocytes. Peaks were ordered based on REC8 enrichment. (B) Diagram showing SYCP1 distribution along genic regions in adult spermatocytes. (C) Putative binding motifs enriched in SYCP1 sites by HOMER algorithm.


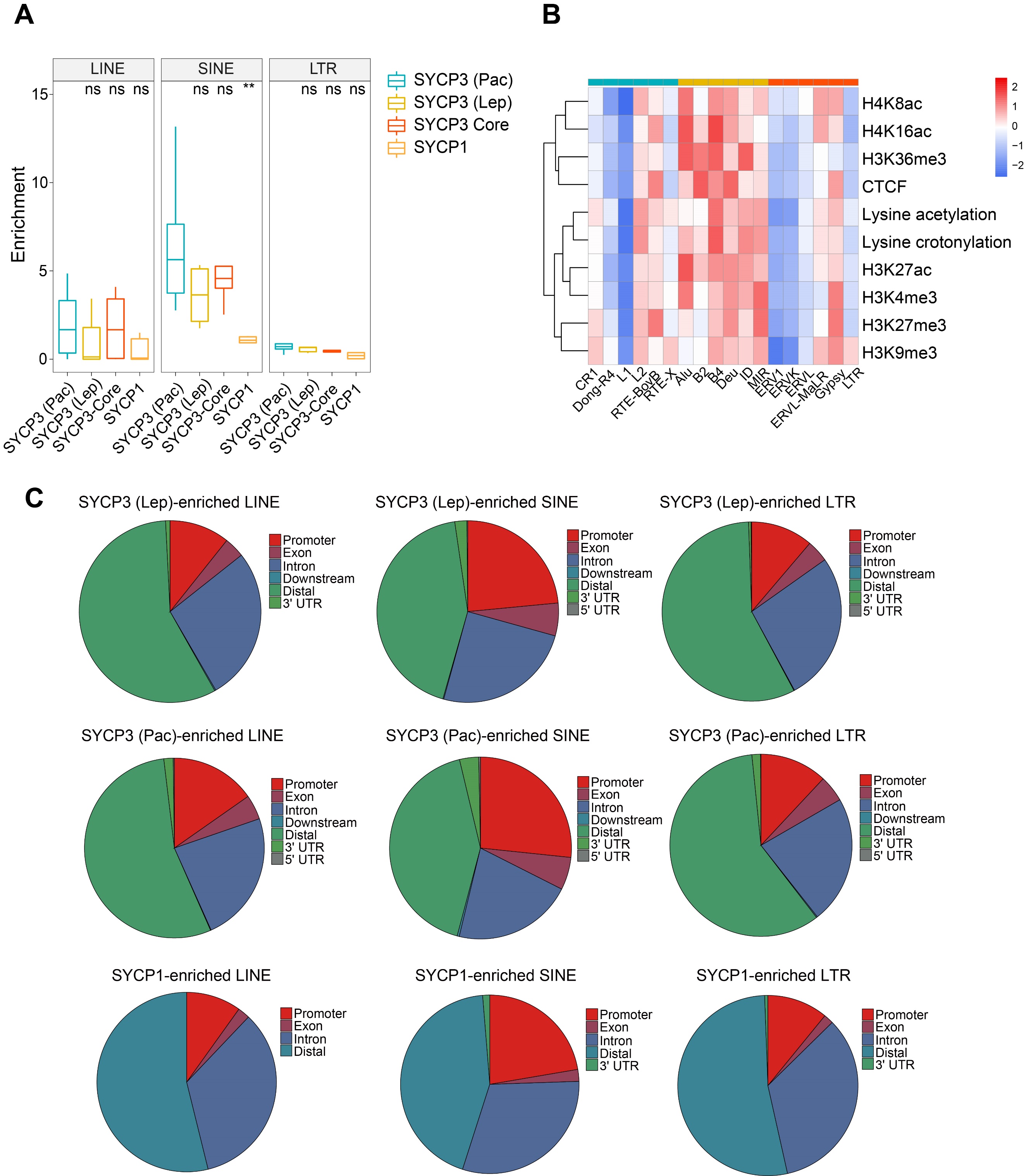


**Supplementary Figure 6. The enrichment of TEs in SYCP3 and SYCP1 occupies regions.**

(A) Box plot showing the enrichment of TE classes in SYCP3 (Pac), SYCP3 (Lep), SYCP3-Core and SYCP1 peaks. (B) Heatmap showing the enrichment and clustering of histone modifications and CTCF at genomic sequences of different TE families. (C) Pie plots showing genomic features of TE family genes with SYCP3 (Lep), SYCP3 (Pac) or SYCP1 peaks.


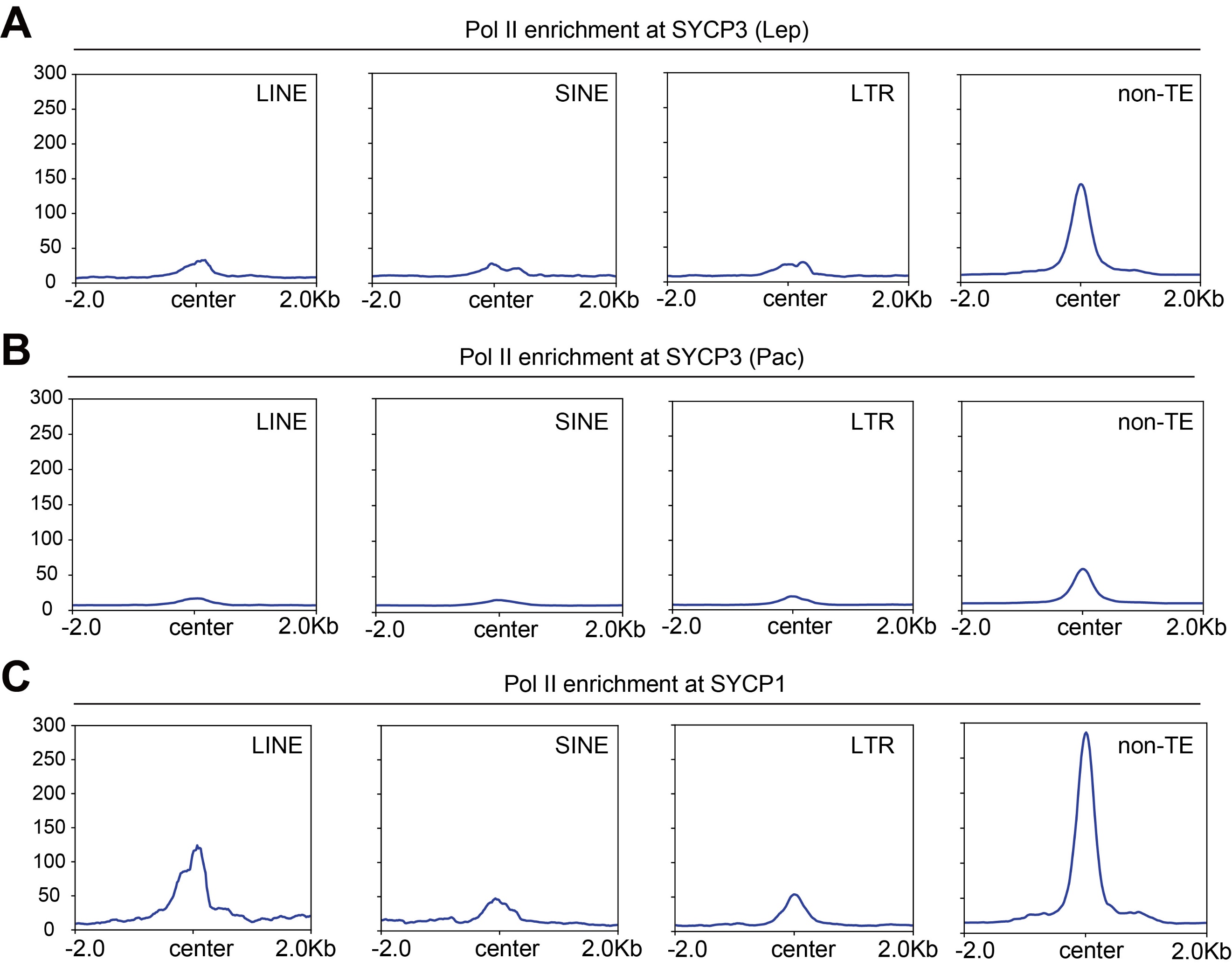


**Supplementary Figure 7. The enrichment of Pol II in SYCP3- and SYCP1-enriched repetitive element regions.**

(A-C) Density plot showing intensities of Pol II at SYCP3 (Lep), SYCP3 (Pac) or SYCP1 peaks, with peaks classified by their colocalization with TE families (LINE, SINE and LTR). Non-TE represents peaks without colocalization with TE loci.


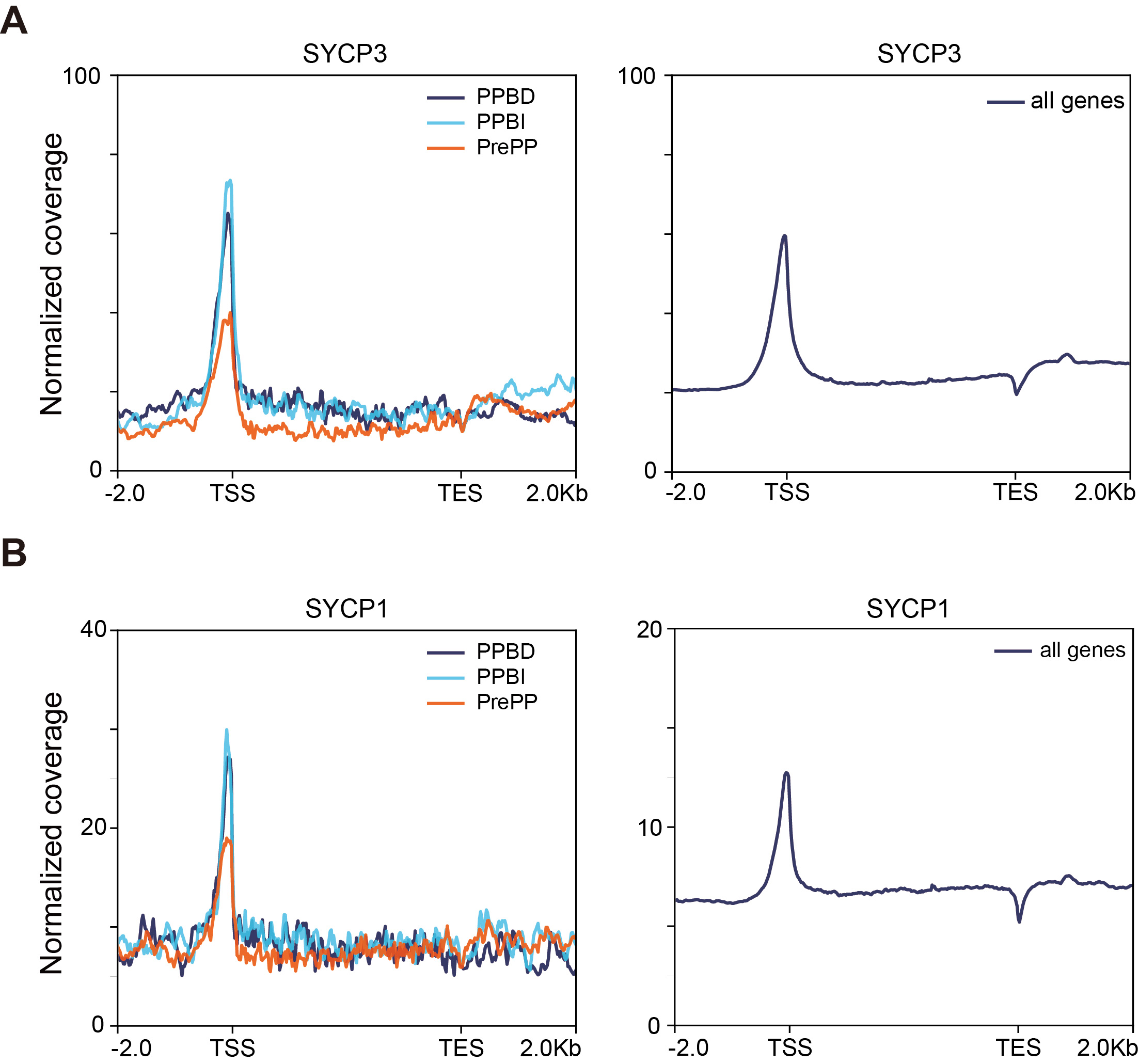


**Supplementary Figure 8. Binding of SYCP3 and SYCP1 at piRNA loci.**

(A) Density plot showing enrichment of SYCP3 at genomic loci of PPBD, PPBI, PrePP and regular genes. (B) Density plot showing enrichment of SYCP1 at genomic loci of PPBD, PPBI, PrePP and regular genes.


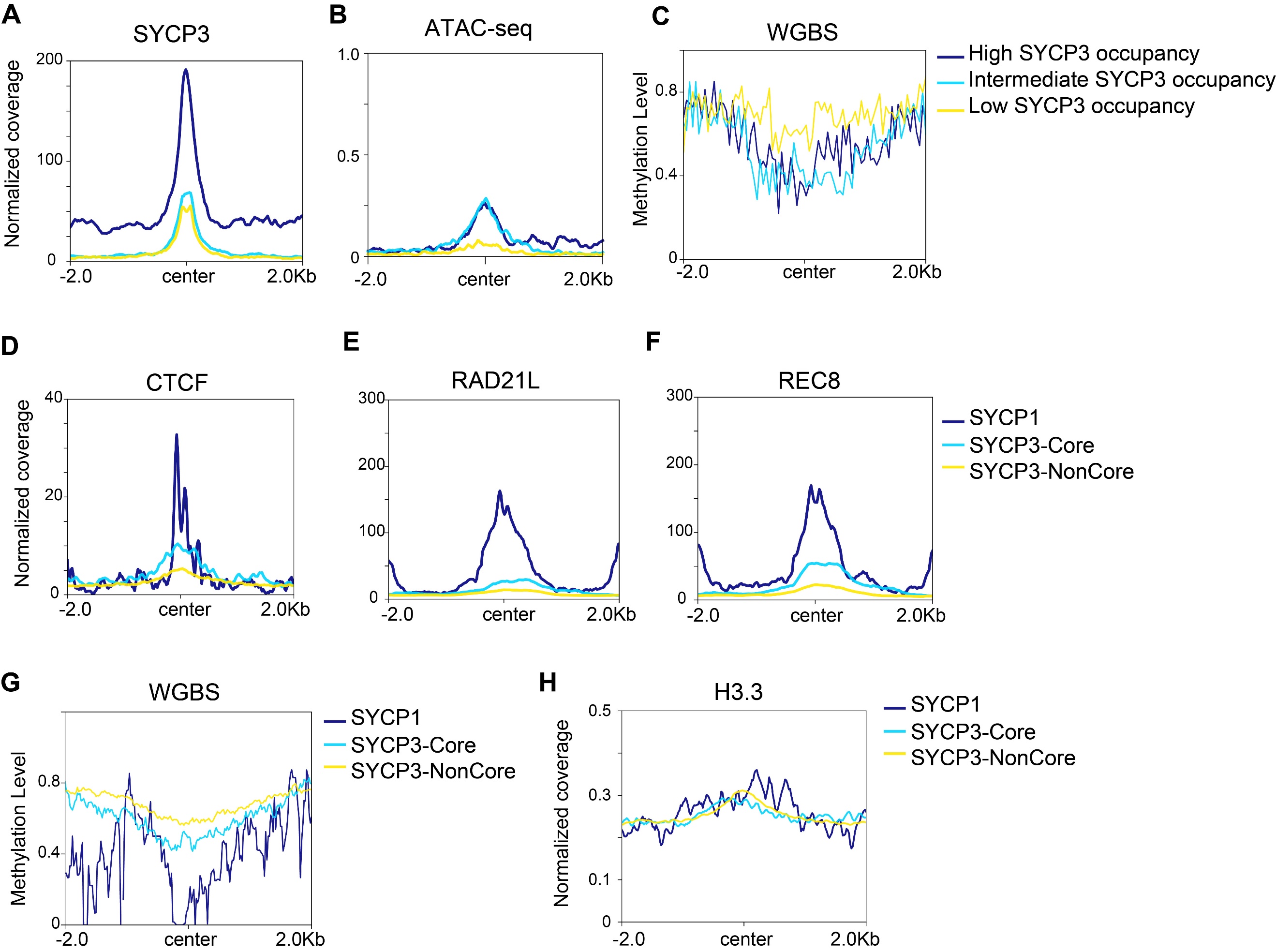


**Supplementary Figure 9. Analysis of SYCP3/1-enriched regions on the sex chromosomes in spermatocytes.**

(A) Density plot showing the distribution of SYCP3 at sex chromosome regions with high/intermediate/low SYCP3 occupancy (peak center ± 2.0 kb). (B-C) Chromatin accessibility and DNA methylation level of SYCP3 peaks with high/intermediate/low SYCP3 occupancy at sex chromosomes in pachytene spermatocytes by ATAC-seq and WGBS. (D-F) Intensity of chromatin association of CTCF (D), RAD21L (E) and REC8 (F) at SYCP3-Core, SYCP3-NonCore and SYCP1 peaks (2 kb flanking center) at sex chromosomes of pachytene spermatocytes. (G) DNA methylation level of SYCP3-Core, SYCP3-NonCore and SYCP1 peaks at sex chromosomes in pachytene spermatocytes by WGBS. (H) Intensity of H3.3 at SYCP1 and SYCP3-Core, SYCP3-NonCore peaks at sex chromosomes in pachytene spermatocytes.


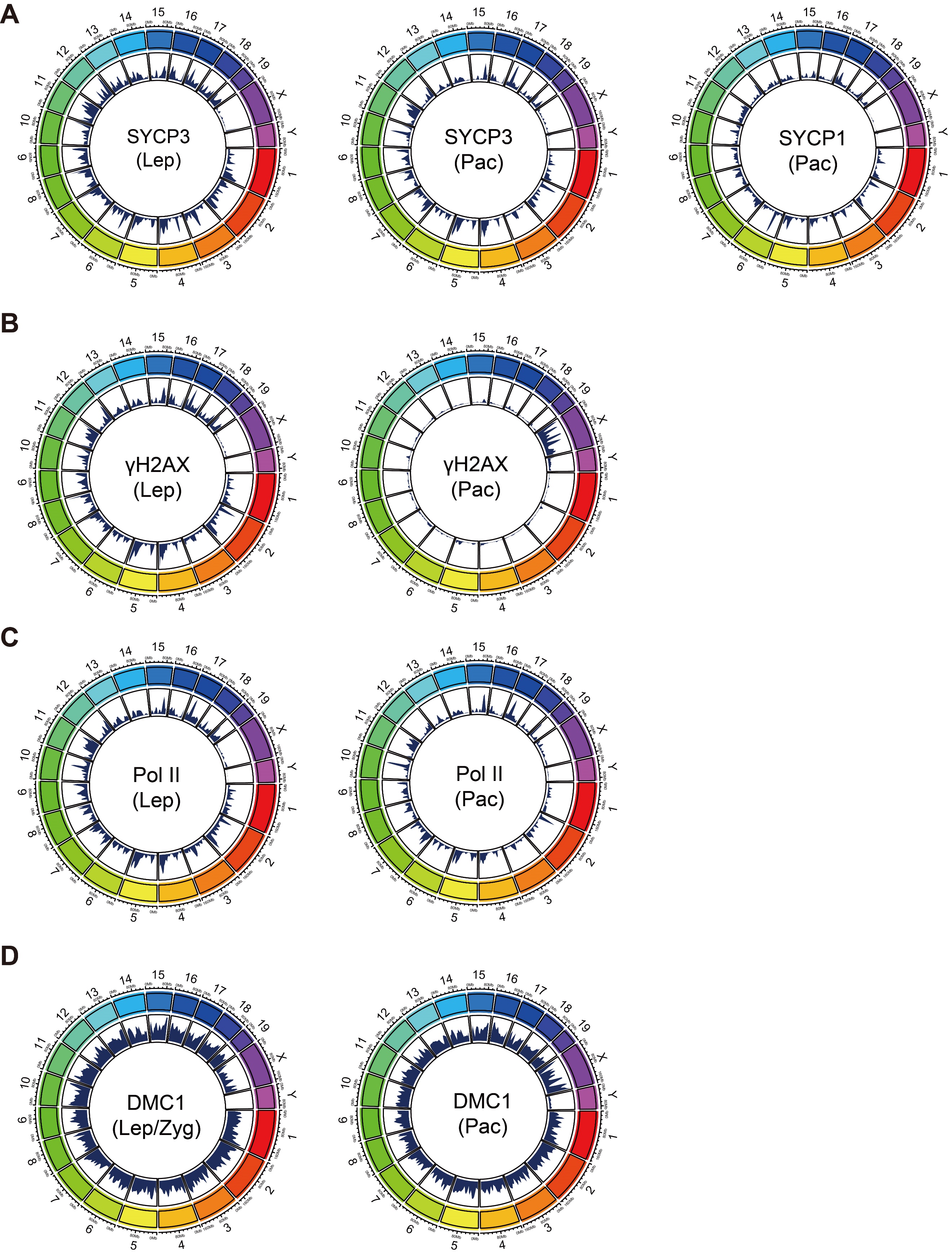


**Supplementary Figure 10. Visualization of peaks along chromosome in spermatocytes.**

(A-D) Circos plots showing distribution maps of mouse chromosomes for SYCP3/1 (A), γH2A.X (B), Pol II (C), DMC1 (D) peaks in mouse spermatocytes.
